# Domestication-driven *Gossypium* profilin 1 (*GhPRF1*) gene transduces early flowering phenotype in tobacco by spatial alteration of apical/floral-meristem related gene expression

**DOI:** 10.1186/s12870-016-0798-0

**Published:** 2016-05-13

**Authors:** Dhananjay K. Pandey, Bhupendra Chaudhary

**Affiliations:** School of Biotechnology, Gautam Buddha University, Greater Noida, 201310 UP India

**Keywords:** Profilin, Apical meristem determinacy, Flower development, Gene expression, Flowering genes

## Abstract

**Background:**

Plant profilin genes encode core cell-wall structural proteins and are evidenced for their up-regulation under cotton domestication. Notwithstanding striking discoveries in the genetics of cell-wall organization in plants, little is explicit about the manner in which profilin-mediated molecular interplay and corresponding networks are altered, especially during cellular signalling of apical meristem determinacy and flower development.

**Results:**

Here we show that the ectopic expression of *GhPRF1* gene in tobacco resulted in the hyperactivation of apical meristem and early flowering phenotype with increased flower number in comparison to the control plants. Spatial expression alteration in *CLV1*, a key meristem-determinacy gene, is induced by the *GhPRF1* overexpression in a WUS-dependent manner and mediates cell signalling to promote flowering. But no such expression alterations are recorded in the *GhPRF1*-RNAi lines. The *GhPRF1* transduces key positive flowering regulator *AP1* gene via coordinated expression of *FT4*, *SOC1*, *FLC1* and *FT1* genes involved in the apical-to-floral meristem signalling cascade which is consistent with our *in silico* profilin interaction data. Remarkably, these positive and negative flowering regulators are spatially controlled by the Actin-Related Protein (ARP) genes, specifically *ARP4* and *ARP6* in proximate association with profilins. This study provides a novel and systematic link between *GhPRF1* gene expression and the flower primordium initiation via up-regulation of the *ARP* genes, and an insight into the functional characterization of *GhPRF1* gene acting upstream to the flowering mechanism. Also, the transgenic plants expressing *GhPRF1* gene show an increase in the plant height, internode length, leaf size and plant vigor.

**Conclusions:**

Overexpression of *GhPRF1* gene induced early and increased flowering in tobacco with enhanced plant vigor. During apical meristem determinacy and flower development, the *GhPRF1* gene directly influences key flowering regulators through *ARP*-genes, indicating for its role upstream in the apical-to-floral meristem signalling cascade.

**Electronic supplementary material:**

The online version of this article (doi:10.1186/s12870-016-0798-0) contains supplementary material, which is available to authorized users.

## Background

Modern crop species are the incredible outcome of the selection force applied on wild plant species by millennia of human-mediated selection, termed as plant domestication. Such evolutionary processes entail events and phenomenon at morphological and genetic levels those have led to certain *morpho*-transitions in the crop species. Such traits consist of a reduction in grain shattering and seed dormancy in cereals [1, 2]; increased apical dominance in maize [3]; increase in seed and pod size, day- neutral flowering in pulses [4, 5]; increased fiber length and quality in cotton [6–8]; increased fruit size in tomato [9]; shorter stolons and larger tubers of potato [10] and many others. Comparative genomics of such acquired phenotypes had increased our understanding of the key genes and *trans*-factors underlying morphological variations in the antecedents and their descendants. For example, *Ghd7* transcription factors in rice for grain number, plant height, flowering time [11]; *tb1* transcriptional regulator and *ZmCCT* gene in maize for plant architecture [12, 13]; *Vrs1* gene in barley for inflorescence architecture [14]; *Sh1* transcriptional regulator in *Sorghum* for non-shattering traits [15] and *HaFT1* transcriptional regulator in Sunflower for improved flowering time [16]. Such information has provided obvious clues for the evolutionary signatures of such morphological characters evolved under crop domestication.

To understand the genetic basis of plant domestication, system-wide comparative gene expression analyses have been performed at different developmental stages of cotton fiber cells harvested from wild and domesticated forms of modern allopolyploid species (*Gossypium hirsutum* L.) [17]. A large number of genes showed differential up- or down-regulation during fiber development. Interestingly, prolonged fiber growth in the domesticated cotton was associated with enhanced hormone signalling genes, delayed stress-responsive gene expression and predominantly modulation of cell-wall structural genes [18]. The latter has drawn much scientific attention to their role in the cell-wall organization, cellular growth and plant development [19]. The dynamic rearrangement of actin filaments is a prerequisite for proper cell wall development because different cell wall proteins work in concordance to maintain stability between filamentous and monomeric actin. It is evident that cell-wall structural protein, especially members of the profilin gene family show up-regulation of at least 400 folds in the domesticated diploid and allotetraploid cotton species than their respective ancestral wild counterpart.

Profilin genes belong to a multigene family extensively diversified across plant species [20–23]. There are five profilin genes *PRF1*, *PRF2*, *PRF3*, *PRF4*, and *PRF5* present in *Arabidopsis* [20, 24, 25]; three members in tobacco [26]; five members in maize [21, 23]; five members in parsley [27]; six members in cotton [19]. Plant profilin is a small cytosolic protein composed of 129–133 amino acids with a low molecular mass of 12-15 kDa that binds to actin monomer in 1:1 complex [21]. Various studies have revealed conserved functions of profilins ranging from lower to higher eukaryotes. In yeast, the profilin genes are involved in cell wall maintenance via actin sequestering, nucleation and cytokinesis [28, 29].

In response to the endogenous or external signals, the cytosolic actin protein undergoes profilin-mediated polymerization and/or depolymerization in a synchronized manner resulting in the cytoskeleton modulation. Profilin promotes the actin-filament formation upon polymerization of sequestered actin monomers present in the cell [30]. In *Drosophila*, profilins are necessary for actin polymerization and its localization throughout stages of development [31, 32]. Profilin genes have also been characterized for their essential roles in plants, such as in pollen formation in maize [23] and tomato [33]; in root nodule development of bean [34]; in cell elongation, cell shape maintenance, and flowering of *Arabidopsis* [24, 35] and in initiation and elongation of cotton fiber [19]. At the molecular level, profilin proteins contribute to the assembly and activity of macromolecular complexes such as polyphosphoinositides [36, 37], Arp2/3 complex [38], annexin [39], prolin-rich ligands [40] and regulating the cell signalling in vivo [41]. So, it determines the key morphological and anatomical traits during plant growth and development.

Several genes are involved in floral induction and flower development. This extraordinarily complex mechanism of flowering is controlled by a number of parallel and/or overlapped pathways governed by diverse genetic networks. Floral meristem identity genes such as *AP1* [42], *AP2* [43], *LFY* [44]; floral pathway integrators such as *SOC1* [45, 46], *FT1* [47] and *FLC1* gene [48] strongly influence the flowering mechanism in plants. The *FT* m-RNA/proteins are synthesized in leaves and get transferred to shoot apex that in result induces expression of downstream flowering genes at apex mainly through binding with *FD* transcription factors [49]. Eventually, it converts the apical shoot meristem into flowering meristem and induces flowering [49, 50]. The *FT* gene is repressed by *EARLY FLOWERING6* (*ELF6*) gene and delays the flowering process in *Arabidopsis* [51]. In general, the floral transition is repressed by *flowering locus C (FLC)* gene that negatively regulates the genes involved in floral pathway integrators [52]. The *RELATIVE OF EARLY FLOWERING 6* (*REF6*) suppresses the expression of *FLC* gene and promotes flowering [53].

However, information is scarce about the involvement of profilin in the flowering mechanism. Profilin is a multifunctional protein and its overexpression in plants results into longer roots and root hair, expanded leaf surface area, accelerating the commencement of flowering in *Arabidopsis* [35, 54, 55], elongated cells in transgenic tobacco culture [56] and early progression of developmental phase in the cotton fiber [57]. Whereas under-expression of profilin gene exhibits smaller phenotype, with at least 40 % reduction in the number of leaves. Reduced expression levels of profilin in *Arabidopsis* delayed initial germination rate and development of seedlings [58]. Defects in rosette leaf morphology and inflorescence stature were reported in response to the lack of *PRF1/PRF2* gene expression in *Arabidopsis* [59]. However, details of the profilin interaction with other genes and *trans*-factors during flowering are to be explored *hitherto*.

Plant profilins have their conventional role in actin polymerization and depolymerization in vivo. Notwithstanding striking discoveries of the genetics of the cell-wall organization in plants [60], limited information is available on the molecular function of profilin and its corresponding network during cell-signalling in the developing apical/floral-meristem. In the present study, we aim to investigate the role of profilin and its molecular interaction considered important in the apical meristem identity and differentiation. This is possible through the dynamic expression interactions among key positive and negative regulators of flowering time phenotypes. Our approach was to use transgenic stocks for up- and down-expression of profilin gene in tobacco; and functional characterization of profilin during flower induction and development. Expression dynamicity of key genes/factors was noted, illuminating possible changes induced in the developmental programs in response to altered profilin levels of the cell. This approach is very useful as a preamble to identify candidate genes interacting with profilin structural protein determining the apical meristem architecture, and governing several other important phenotypic traits during plant development.

## Results

### Constitutive overexpression of cotton profilin 1 (*GhPRF1*) in tobacco shows early flowering

**Fig. 1:**
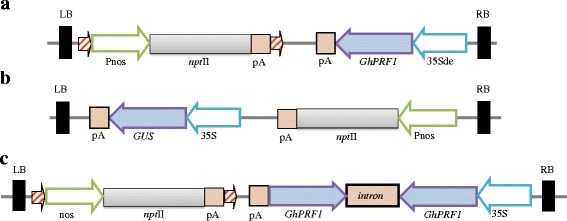
Schematic representation of different gene constructs used in the present study. **a** The T-DNA diagramme of nos:*nptII*:pA::CaMV35S:*GhPRF1*:pA binary construct for the overexpression of proflin gene in tobacco. **b** The T-DNA diagramme of nos:*nptII*:pA::CaMV35S:*gus*:pA binary construct. **c** The T-DNA diagramme of RNAi binary construct nos:*npt*II:pA::CaMV35S:*GhPRF1-intron-GhPRF1*:pA gene construct for the down-expression of proflin gene in tobacco. The orientations of different gene cassettes are shown as per their respective cloning sites in the binary vector. The horizontal bars are not to scale

Previously, we had explored the evolution of global gene expression patterns under cotton domestication through comparative transcript profiling experiments performed on the fiber cells harvested at three developmental stages from wild and domesticated accessions of allopolyploid *Gossypium barbadense* and *G. hirsutum*, using a microarray platform that interrogates 42,429 unigenes [17, 18]. Notably, cell-wall related profilin gene family is one of the structural gene families that has been highly up-regulated parallelly and independently in the domesticated accessions of both allopolyploid species in contrast to their respective wild counterparts. To study the role of cotton profilin structural genes in other plant phenotypes, the spatial expression analysis was performed in ectopically expressed profilin transgenic tobacco lines. The full-length *GhPRF1* gene (accession number EF143832) consisting of 402 bp was constitutively expressed under the control of Cauliflower Mosaic Virus 35S (CaMV35S) promoter with double enhancer region (35Sde) in tobacco (*Nicotiana tabacum* cv. Xanthi), along with *nos*:*npt*II:pA gene cassette as a plant selection marker (Fig. 1a). The 35Sde comprises of a repeat of the −90 to −343 region of the 35S promoter upstream of the wild- type 35S promoter that functions as an enhancer [61]. Along with two more binary constructs having *GhPRF1* gene and *gus*-gene were developed for the generation of *gus*-reporter lines and RNAi lines, respectively having *npt*II gene as plant selection marker (Fig. 1b, 1c). Using leaf explants, *Agrobacterium*-mediated genetic transformation of tobacco was performed and twenty seven independent transgenic lines with 35Sde-*GhPRF1* gene were developed. These independent lines were screened for the transgene integration through PCR using *npt*II specific primers. The PCR positive transgenic lines along with in vitro regenerated control Xanthi plants were simultaneously transferred to the controlled growth conditions.

**Fig. 2:**
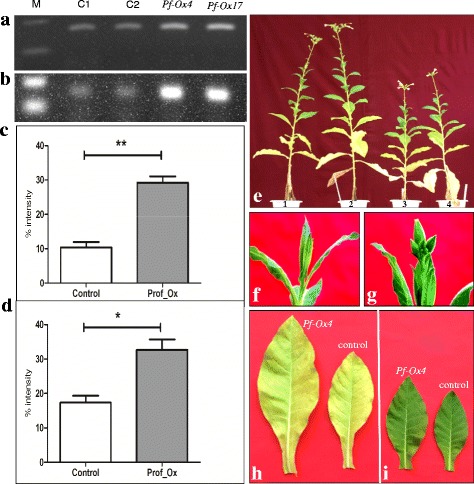
Profilin gene expression analysis in vegetative and reproductive tissues of two transgenic overexpression lines *Pf-*Ox4 and *Pf-*Ox17 of tobacco. **a** Semi-quantitative expression analysis of profilin in vegetative tissues of *Pf-*Ox4 and *Pf-*Ox17 in comparison to two independently in vitro regenerated control lines. **b** Semi-quantitative expression analysis of profilin in reproductive tissues of *Pf-*Ox4 and *Pf-*Ox17 in comparison to two independently in vitro regenerated control lines. **c** & **d** Average expression values of profilin gene in both vegetative and reproductive tissues of the two overexpression lines in comparison to control lines, respectively, by densitometry imaging analysis avoiding any biases visible in the band intensities on an agarose gel. **e** Significant increase in the plant height through elongated internodal regions of over-expression lines *Pf-*Ox4 and *Pf-*Ox17 (labeled 1 & 2) in comparison to two control lines (labeled 3&4) after 120 dpt. **f** In vitro regenerated control line after 100 days of vegetative growth post-transplantation. **g** Transgenic *Pf-*Ox4 line showing early conversion of apical shoot meristem into floral meristem after 100 days of vegetative growth post-transplantation. **h** Relative size differences in 20^th^ leaf from the top of transgenic and control lines. **i** Relative size differences in 13^th^ leaf from the top of transgenic and control lines

In semi-quantitative expression screening of PCR positive lines, significant over-expression of *GhPRF1* gene were recorded in several transgenic lines including two lines *Pf*-Ox4 and *Pf*-Ox17 that showed a significant increase in the profilin transcript level compared to the control plants (Fig. 2a, 2b). Transgenic lines *Pf*-Ox4 and *Pf*-Ox17 showed more than 20 % increase in the *GhPRF*-transcript level in the leaf tissues and up to 17 % up-regulation in the reproductive organs (Fig. 2c, 2d). The two high *GhPRF1*-transgene expression lines exhibited developmental phenotypes assessed at different stages of plant development (Fig. 2e). Such qualitative phenotypic changes were similar in both the transgenic lines.

### Ectopic expression of *GhPRF1* leads to the hyperactivation of apical meristem and alters spatial expression of meristem-related *CLAVATA1* gene

The complimentary DNA of *GhPRF1* was expressed under the control of CaMV35S promoter in Xanthi. After transgene-based screening of putatively transformed lines, one of the most significant changes examined in both transgenic lines *Pf*-Ox4 and *Pf*-Ox17 was the hyperactivation of apical meristem after at least 50 days of vegetative growth post-transplantation (dpt) in the soil and its further differentiation into floral meristem at 99-100 dpt than control plants at 110-112 dpt (Fig. 2f, 2g). To ascertain if the enhanced apical growth and its early conversion into floral meristem were due to faster apical-meristematic cellular division and differentiation, elongation of internodal regions and conversion rate of apical-to-floral meristem were determined for representative *Pf-*Ox4 and *Pf*-Ox17 overexpression lines. The overexpression lines resulted in the significant increase in the organ (leaf) differentiation and expansion than control lines (Fig. 2h, 2i). The apical shoot meristem was converted into floral meristem earlier in *Pf*-Ox4 and *Pf*-Ox17 overexpression lines than control plants resulting into increased number of fully developed flowers. These differences in the developmental conversion of apical meristem can be attributed, at least in part, to the overexpression of *trans*-*GhPRF1*.

Further, to identify temporal alterations in the regulation of a complex genetic-network of apical meristem activity in *GhPRF1* overexpression lines, leaf tissues were harvested at the similar developmental time points avoiding physiological variations between transgenic and control lines. Similarly, floral bud tissues were harvested from both transgenic and control lines. Comparative expression analyses of selected genes *CLAVATA1* (*CLV1*) and *WUS* were performed in both *Pf*-Ox4 and *Pf*-Ox17 transgenic lines along with control plant. These genes/*trans*-factors have been reported for their direct role in the regulation of meristem identity, maintaining a balance between cell proliferation and organ formation at shoot/flower meristems (Table 1).

**Table 1:** Genes and *trans*-factors underlying shoot meristem to flower transition during plant development

S.N.	Genes	Types	Functions	References
1	*KNOX*	Transcription factor	Regulation of meristem identity in plants (monocots and dicot); Activation/repression of GA-synthesis genes	[95, 96]
2	*CLAVATA1(CLV1)*	Receptor-like kinase	Maintenance of an equilibrium between cell enlargement and organ development; Regulation of apical/floral meristem determinacy	[76, 97]
3	*CLAVATA 3 (CLV3)*	Peptide	Binding with the receptor-like kinase *CLV1* as its ligand; Restricts *WUS* expression	[65, 76]
4	*AUXIN-BINDING PROTEIN 1 (ABP1)*	Auxin binding protein	ABP1- TMK1 complex formation for auxin perception; Maintenance of asymmetric growth at floral primordial region	[75, 99]
5	*RHO OF PLANTS 6 (ROP6)*	Plasma membrane associated small GTPase	Regulation of cellular processes; Maintenance of asymmetric growth under the influence of auxin	[75]
6	*SEPALLATA 3 (SEP3)*	MADS-domain transcription factor	Interaction with floral genes *AG* and *AP3*; Regulation of floral organ formation	[98]
7	*ROP-INTERACTIVE CRIB MOTIF-CONTAINING PROTEIN 1 (RIC1)*	CRIB-containing ROP effector	Regulator of ROP6 gene; Maintenance of asymmetric growth under the influence of auxin	[99]
8	*APETALA 1 (AP1)*	MADS-domain transcription factor	Chromatin remodelling; Regulation of flower initiation	[74, 98]
9	*WUSCHEL (WUS)*	Homeodomain transcription factor	Stem cell activity in meristematic regions; Regulation of floral meristem determinacy	[65]
10	*LEAFY (LFY)*	Transcription factor	Regulation of floral meristem identity; Activator of *AG, * *AP3 *and *AP1 *genes	[73, 74]

**Fig. 3:**
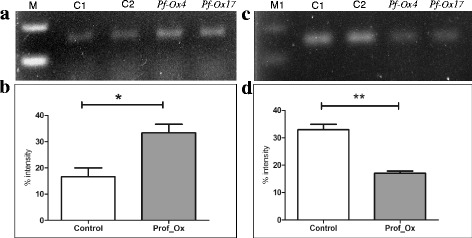
*CLV1* gene expression analysis in vegetative and reproductive tissues of two transgenic overexpression lines. **a** Semi-quantitative expression analysis of *CLV1* gene in vegetative tissues of *Pf-*Ox4 and *Pf-*Ox17 in comparison to two control lines (C1 and C2). **b** Semi-quantitative expression analysis of *CLV1* gene in reproductive tissues of *Pf-*Ox4 and *Pf-*Ox17 in comparison to the control lines. **c** & **d** Average expression values of *CLV1* gene in both vegetative and reproductive tissues of the two overexpression lines in comparison to control lines, respectively, by densitometry imaging analysis avoiding any biases visible in the band intensities on agarose gel

**Fig. 4:**
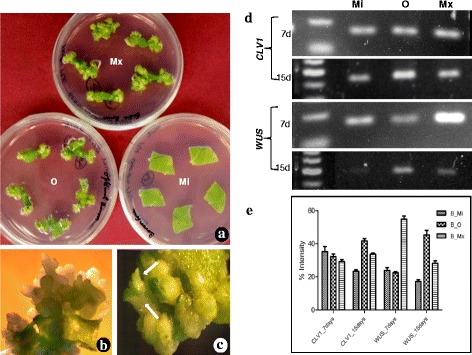
**a** In vitro organogenesis of tobacco on MS medium supplemented with NAA (0.01 mg/l) and BAP (1.0 mg/l) phytohormones. Three different concentrations i.e., minimal (Mi; <0.1 mM), optimal (O; 0.1 mM) and maximal (Mx; >0.1 mM) of micro-nutrient Boron was supplied with MS medium and leaf explants were cultured. **b** Induction of shoot primordia on the edges of leaf explants after15 days of culture on MS shoot induction medium. **c** An enlarged view of a microscopic shoot meristem (shown by an arrow) which was harvested for meristem-determinacy gene expression analysis. **d** Temporal expression of *CLV1* gene analyses in 7 days and 15 days old shoot primordia harvested from explants cultured on minimal, optimal and maximal boron- supplemented medium. Similarly, semi-quantitative expression of *WUS* gene was analysed in 7 days and 15 days shoot primordia harvested from explants cultured on minimal, optimal and maximal boron- supplemented medium. **e** Average expression values of *CLV1* and *WUS* genes in shoot primordia after 7 days and 15 days of culture, measured by densitometry imaging analysis avoiding any biases visible in the band intensities on an agarose gel

In semi-quantitative expression study, the endogenous transcript level of *CLV1* receptor-kinase gene varied enormously across developmental stages. *CLV1* gene encodes a putative receptor-like kinase which plays an important role in signal transduction during shoot induction. Expression of *CLV1* results into the formation of shoot primordia and increases the proliferation of undifferentiated mass of cells destined for shoot formation. Therefore, it was decided to examine the expression levels of *CLV1* receptor-kinase gene in both vegetative and floral tissues. Reverse transcription-PCR was performed to determine if constitutive overexpression of *CLV1* receptor-kinase gene transcript is detectable in both *Pf*-Ox4 and *Pf*-Ox17 transgenic lines. Interestingly, the expression of *CLV1* receptor-kinase gene showed continuous spatial alteration in its expression levels across tissues and developmental stages of both *Pf*-Ox4 and *Pf*-Ox17 overexpression lines (Fig. 3a, 3b). Up to 14 % increase in the expression level of *CLV1* receptor-kinase gene in the vegetative tissue of *Pf*-Ox4 line was observed in comparison to control lines. On the contrary, more than 30 % reduction in the expression level of *CLV1* receptor-kinase gene was observed in the floral tissues of *Pf*-Ox4 and *Pf*-Ox17 lines than vegetative tissues (Fig. 3c, 3d). Based on these findings, profilin-mediated functional polymerization of proteins combined with *CLV1* receptor-kinase is adequate for the activation and determinacy of apical meristem that accomplishes the apical-to-floral meristem conversion. Further, to explore if *CLV1* receptor-kinase is a functional contributor to such an extraordinarily complex process of meristem growth, development and differentiation, in vitro organogenesis experiments were performed in tobacco explants cultured on MS medium [62] supplemented with auxin (NAA; 0.1 mg/l) and cytokinin (BAP; 1.0 mg/l) (Fig. 4a). Previously, in our laboratory the effect of micronutrient Boron (B) has been reported on the magnitude of organogenesis in tobacco when cultured on minimal (<0.1 mM), optimal (0.1 mM) and maximal (1.0 mM) B-concentrations [63]. It was evident that explants expansion and growth was more in minimal B-concentration than other concentrations, however, in vitro shoot induction was the highest at an optimal concentration of B-supplementation (Fig. 4b, 4c). To determine if *CLV1* gene has a certain role in the initiation and progression of shoot development (during organogenesis), the temporal *CLV1* expression was analysed under different B-concentrations in 7 days and 15 days old cultured explants.

Interestingly, no significant change in the basal expression level of *CLV1* gene across B-treatment was observed at 7 days old cultured explants, however, significant alterations were observed at 15 days old tissues (Fig. 4d, 4e). *CLV1* expression was substantially increased in the shoot-buds cultured on optimal B-supplementation than minimal or maximal concentrations. This suggests that *CLV1* expression in shoot-buds is proportionate with the magnitude of organogenesis (shoot induction/formation). This may be assumed that in 7 days old cultures, initially the explants undergo callogenesis followed by organogenesis, after 15 days of the culture period (Fig. 4b). At this stage of development, the callus-foci are converted into shoot primordia and important genes/factors such as *CLV1* gene considered to be accountable for organogenesis are most likely to be expressed at this shoot-bud stage.

### *GhPRF1* overexpression shows enhanced organogenesis in vitro

**Fig. 5:**
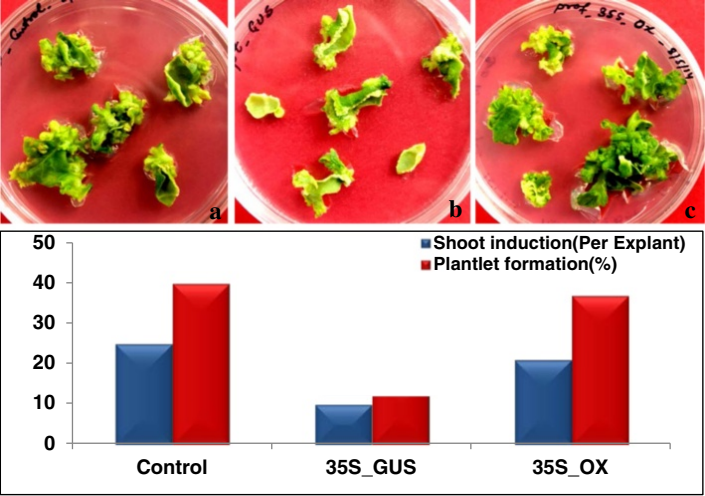
Influence of *GhPRF1* overexpression on the shoot induction (organogenesis). **a** Untransformed Xanthi explants cultured on MS medium supplemented with phytohormones required for shoot induction. **b** Explants transformed with nos:*npt*II:pA::35S:*gus*:pA gene cassettes and cultured on MS medium + Phytohormones + kanamycin (100 mg/l). **c** Explants transformed with nos:*npt*II:pA::35:*GhPRF1* gene cassettes and cultured on MS medium + Phytohormones + kanamycin (100 mg/l). The lower panel shows significant changes in the rate of organogenesis and % plantlet formation in *GhPRF1* transformed explants in comparison to 35S:*gus* transformed explants and untransformed explants

Given that the *CLV1* expression was radically increased in the emerging shoot buds in vitro, what would be the magnitude of organogenesis in the shoot buds transformed with *GhPRF1* gene intended to enhance *CLV1* expression? Since in vitro genetic transformation experiments were carried out on kanamycin (100 mg/l) antibiotic selection marker that may negatively influence organogenesis, genetic transformation experiments with 35S-*gus* construct were performed parallelly, therefore avoiding detrimental effects of kanamycin on the magnitude of shoot induction in 35Sde-*GhPRF1* transformed explants (Fig. 1b). This was bolstered in the genetic transformation experiments using *GhPRF1* transgene where shoot formation was observed up to 37 % in 35Sde-*GhPRF1*, 23 % in 35S-*gus* transformed explants and 29 % in optimal B-supplemented media, of the inoculated leaf explants (Fig. 5). This observation suggested that *GhPRF1* overexpression directed the up-regulation of *CLV1* expression during apical meristem initiation.

### RNAi of *GhPRF1* showed delay in flowering and reduced flower number than overexpression lines

**Fig. 6:**
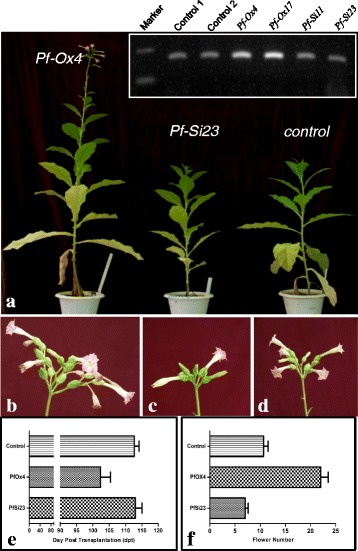
Genetic manipulation of profilin gene in tobacco. **a** Transgenic plants are developed with ectopic constitutive overexpression line, constitutive down-expression line, and control line, respectively. All three lines are photographed at the same age. The inset picture shows the relative expression of proflin gene in control, overexpression, and RNAi silencing line. **b** Flower number per plant is shown for *Pf-*Ox4 line. **c** Flower number per plant is shown for the *Pf-*Si23 line. **d** Flower number per plant is shown for control line. **e** A number of flowers produced by *Pf-*Ox4 and *Pf-*Si23 transgenic lines along with control plant. One way ANOVA analysis was performed for statistical analysis of differences using Graphpad Prism resulted in R^2^ value 0.9467. **f** The onset of flowering in *Pf-*Ox4 and *Pf-*Si23 transgenic lines along with control plant after transplantation. Unpaired *t*-test was performed (*p* < 0.05) to analyze significant differences in flowering time

To determine, if early flowering time phenotype as shown by *Pf-*Ox4 and *Pf-*Ox17 transgenic lines was attributed to profilin overexpression, down-expression of *GhPRF1* gene through RNAi was experimented. For this purpose, a *GhPRF1*-RNAi gene construct was developed using pHANNIBAL cloning vector [64] having an intron of 742 bp flanked by inverted and palindromic sequences of full-length profilin gene (Fig. 1c). Using *GhPRF1*-RNAi construct, *Agrobacterium*-mediated genetic transformation of tobacco leaf explants was performed. Twenty-seven transgenic lines were developed and screened for transgene presence with the help of PCR. All PCR positive lines were screened for % *GhPRF1* silencing using RT-PCR; and six independent transgenic lines were identified with substantial down-regulation of profilin expression. In particular, line *Pf-*Si23 showed maximum 36 % down-expression of *GhPRF1* transcript level compared to *Pf-*Ox4 line. At phenotypic level, the plant height of down-expression *Pf-*Si23 line was significantly lower than *Pf*-Ox4 line but similar to the control plants (Fig. 6a). The average number of flowers per *Pf-*Si23 plant was lesser than both *Pf-*Ox4 line and control plants (Fig. 6b, 6c, 6d, 6e). The flowering initiation time of transgenic line *Pf-*Si23 was recorded at least 10 days later than *Pf-*Ox4 line and similar to the control plants (Fig. 6f). Also, delayed conversion of apical shoot meristem to floral meristem and with reduced number of flowers per plant in *Pf-*Si23 line proved that profilin expression controls apical meristem development, differentiation, floral initiation and development through its interaction with other known genetic factors.

### Overexpression of *GhPRF1* regulates floral determinacy by arrested expression of *WUSCHEL trans-*factor

Prompted by an intriguing observation made on the increased expression of *CLV1* receptor-kinase gene in response to overexpression of *GhPRF1* in transgenic *Pf*-Ox4 and *Pf*-Ox17 lines achieving hyperactivation of apical meristem, it was relevant to determine if the expression patterns of other coordinating factor(s) involved in the genetic regulation of floral determinacy show any dynamicity in their expression patterns. As summarized in Table 1, *WUSCHEL (WUS)* homeodomain transcription factor is involved in stem cell activity in the central zone of apical meristem and controls floral determinacy. *WUS* controls the stem cell fate and maintain its expression level by up-regulating the *CLV* gene through feedback regulation [65]. Therefore, the expression analysis of *WUS* gene in both vegetative and floral tissues of both *Pf*-Ox4 and *Pf*-Ox17 lines was performed. It was observed that both *GhPRF1* overexpressed lines showed down-regulation of *WUS* transcripts level in the floral buds than the vegetative tissues. This may be expected that high accumulation of profilin in *Pf*-Ox4 and *Pf*-Ox17 lines arrests *WUS* expression either directly or through coordinated factors, thus adversely influencing the feedback loop during floral determinacy [65].

Further, to determine if *WUS* expression varies in comparison to the magnitude of organogenesis and in coordination with *CLV1* gene expression, the temporal *WUS* expression was analysed in 7 days and 15 days old leaf explants cultured under different B-concentrations. Surprisingly, in maximum B-supplemented callus tissues, at least 3 fold increased expression of *WUS* gene in 7 days old cultures was observed than 15 days old cultures (Fig. 4e). At this stage of culture, the *CLV1* expression is examined at basal level (Fig. 4d) and is congruent with established correlation of the two genes under consideration. On the contrary, in 15 days old cultures (shoot-buds) *WUS* expression level was enhanced up to 1.5 fold in optimal B-supplemented cultures than maximal B-supplemented cultures. This is in concordance with the magnitude of organogenesis reported on optimal B-supplementation in tobacco (unpublished data). At this stage of development, up-regulation of *WUS* leads to the overexpression of *CLV1* gene thus controlling *WUS* at transcript level through feedback and simultaneous progression of shoot primordia at the phenotypic level. Taken together, our work shows that overexpression of profilin down-regulated *WUS* expression level *via* modulated *CLV1* transcript levels during floral initiation and development.

### Dynamicity of coordinated expression patterns of key flowering genes in *GhPRF1* overexpression and silencing lines

**Fig. 7:**
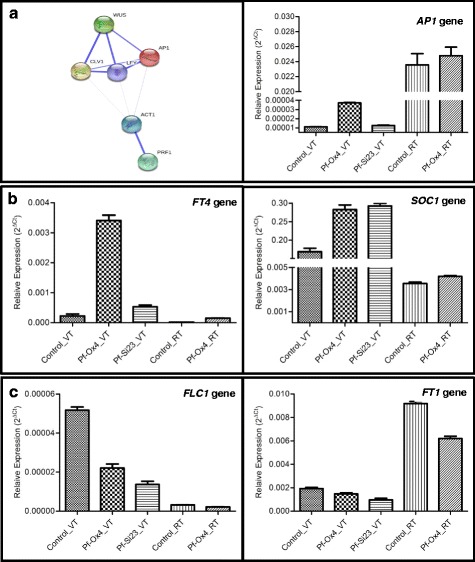
Dynamicity of coordinated profilin interaction network with key flowering regulators predicted *in silico* and analyzed at the transcript level in *GhPRF1* over- expression and RNAi lines. The genes encoding for positive and negative flowering regulators were analysed in vegetative tissue (VT) and reproductive tissue (RT) of control plant, *Pf-*Ox4 overexpression line and *Pf-*Si23 silencing line. **a** *In silico* prediction of profilin interaction with key flowering genes based on STRING 10. This panel also shows the positive flowering regulator *AP1* gene expression data in vegetative tissue (VT) and reproductive tissue (RT) of the control plant, *Pf-*Ox4 line and *Pf-*Si23 line. In the *in silico predicted* interaction map, line thickness represents the confidence level of protein-protein interaction. **b** This panel represents the positive flowering regulators *FT4* and *SOC1* gene expression data in VT and RT of different lines. **c** This panel represents the negative flowering regulators *FLC1* and *FT1* gene expression data in VT and RT of different lines

Since early flowering time phenotype was observed in both *Pf*-Ox4 and *Pf*-Ox17 lines, it is to determine if key positive or negative flowering regulators show any dynamicity in their expression patterns? Coordinated profilin interaction network with key flowering control genes was predicted *in silico* using STRING 10 online tool [66]. The prediction based interaction map suggested distinct protein clusters of key flowering genes and cytoskeleton- related genes (Fig. 7a). High interaction is observed within meristem-related *CLV1*, *WUS* and key flowering genes such as *LFY*, *AP1* and cytoskeleton proteins. However, the low interaction was evident between the two protein clusters (Fig. 7a). Further to determine the dynamicity of such profilin interaction network with flowering regulators predicted *in silico*, the spatial expression of positive flowering regulators *FT4, SOC1* and *AP1* genes [42, 45–47, 49, 50] and negative regulators *FLC* and *FT1* gene [48, 52, 67] was examined in the vegetative and the floral bud tissues of both overexpression and down-expression transgenic lines. The class ‘A’ flowering *AP1* gene showed 3.5 fold increased expression in the vegetative tissue of *Pf*-Ox4 line than control plants or *Pf-*Si23 line (Fig. 7a). This *AP1* gene expression showed at least 700 folds increase in 100 dpt old floral bud tissue than vegetative tissue of *Pf*-Ox4 line, which was similar to 110 dpt old reproductive tissue of control plant (Fig. 7a). This data highlighted the mechanistic link between profilin overexpression and early flower primordium initiation via up-regulation of *AP1* gene. Similarly, *FT4* gene, a positive flowering regulator which is initially synthesized in the leaves and travels to apical meristem showed more than 16 folds increase in its expression in the vegetative tissues of *Pf*-Ox4 line than *Pf-*Si23 line and control plants (Fig. 7b). Whereas in the reproductive tissues of *Pf*-Ox4 line and control plant, *FT4* expression decreased drastically, highlighting its role in the up-regulation of flowering regulators *SOC1*, *LFY* and *AP1 *genes. These genes are responsible for the activation of class ‘B’ genes during floral development. In response to elevated *FT4* expression in the vegetative tissue of *Pf*-Ox4 line, homeodomain *SOC1* transcription factor showed at least 1.5 fold increase in its expression level than control plant (Fig. 7b). However, *SOC1* expression in *Pf-*Ox4 line was similar to the *Pf-*Si23 line which showed that the up-regulation of *SOC1* transcription was intermediate to the flowering mechanism for up-regulation of *AP1* gene. Whereas no significant difference in *SOC1* expression was observed in the reproductive tissues of *Pf*-Ox4 lines and control plant (Fig. 7b). Therefore, early flowering time phenotype is controlled by the molecular interaction of up-regulated *FT4* gene with *SOC1* and *AP1* genes. This is further confirmed by the *Pf*-Si23 line which showed flowering similar to control plants despite the up-regulation of positive regulator *SOC1* gene. This observation confirmed the central role of profilin in early flower primordium formation via up-regulation of *AP1* gene. Conversely, negative flowering regulators *FLC* and *FT1* genes in the vegetative tissues of overexpression line in comparison to the control plant showed at least 2.5 and 1.18 fold decrease in their respective expression levels. Whereas *FT1* gene which is a negative regulator of flowering in tobacco [67] showed 1.5 fold reduced expression level in the reproductive tissues of *Pf*-Ox4 line compared to the control plant (Fig. 7c). This coordinated expression patterns of both negative and positive regulators in the vegetative tissues are contemplated to advance the cellular milieu for flower induction. Since *Pf*-Si23 line showed flowering time similar to control plants, the expression patterns of positive regulators *AP1*, *SOC1* and *FT4* were analysed in the floral buds of *Pf*-Ox4 and *Pf*-Ox17 lines. *FT4*, *SOC1* and *AP1* genes showed coordinated overexpression in both *Pf*-Ox4 and *Pf*-Ox17 lines, whereas key negative regulators *FLC* and *FT1* genes exhibited significant down-regulation across tissues and stages (Fig. 7c). Thus, on the acquisition of flower induction in both *Pf*-Ox4 and *Pf*-Ox17 lines, the overall coordinated regulation of positive and negative flowering regulators in vegetative and floral buds is remarkable.

### *GhPRF1* acts upstream to the apical-to-floral meristem signalling cascade *via* coordinated expression of *ARP4* and *ARP6* genes

**Fig. 8:**
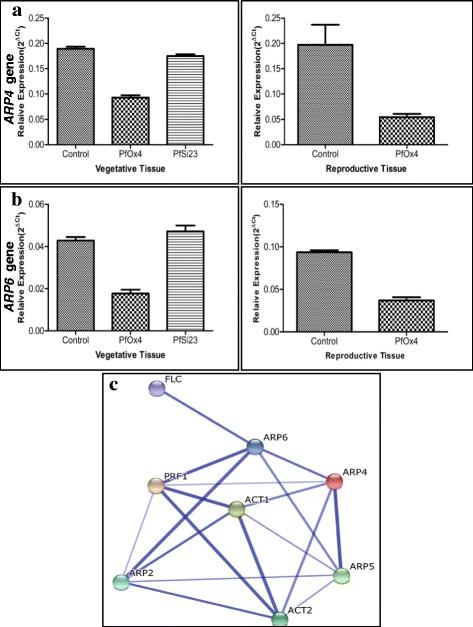
Gene expression analyses of *ARP* genes and prediction of their interaction with other genetic factors. **a** *ARP4* gene expression analyses in *Pf-*Ox4 and *Pf-*Si23 transgenic lines along with control plant in VT and RT tissues. **b** *ARP6* gene expression analyses in *Pf-*Ox4 and *Pf-*Si23 transgenic lines along with control plant in VT and RT tissues. **c** *In silico* prediction of profilin-*ARP* interaction based on STRING 10. This analysis shows the interaction of profilin with actin proteins and one of the key flowering regulator FLC *via* ARP6 protein. The line thickness represents the confidence level of protein-protein interaction

To further examine the role of profilin upstream to the coordinated spatial regulation of flowering regulators in the overexpression lines, we investigated the cellular and molecular effectors including *ABP1*, *PIP*, and *ARP* genes involved in the apical-to-floral meristem signalling cascade. The expression patterns of *ABP1*, *PIP*, and *ARP* genes (especially *ARP4* and *ARP6* known for their defined function in flower development; Table 2) were analysed in the vegetative and floral bud tissues of both the overexpression and down-expression transgenic lines. Remarkably, *ARP4* gene showed 2.3 fold reduced expression in the vegetative tissue of *Pf*-Ox4 line than RNAi line and control plant (Fig. 8a). Subsequently, *ARP4* gene expression showed at least 4-fold decrease in floral bud tissues of *Pf*-Ox4 line than the similar tissues of control line which was at least 2 fold less than the respective vegetative tissue (Fig. 8a). Consistently, *ARP6* gene, another profilin-associated flowering regulator showed 2.5 fold down-expression in the vegetative tissue of *Pf*-Ox4 line; and 1.2 fold increased expression in *Pf*-Si23 RNAi line than the control plant (Fig. 8b). Whereas in reproductive tissues of the *Pf*-Ox4 line, more than 2 fold down-expression of *ARP6* gene was observed than the control plant (Fig. 8b). Overall, *ARP4* and *ARP6* genes showed down-expression in their expression level exclusively in the overexpression lines. However, no such variations in the spatial gene expression pattern of *ABP1* and *PIP* genes were observed among transgenic lines. This data showed that overexpressed profilin up-regulates flowering genes expression cascade by regulating *ARP4* and *ARP6* gene expression which in result influence *FLC1* gene and flower induction. Further, coordinated profilin interaction network including key cytoskeletal genes such as actin, and *ARP*s was predicted *in silico* using STRING 10 online tool [66]. The prediction map suggested significant interaction among profilin, actin, and ARPs especially with ARP4 and ARP6 with high confidence limits (>0.900) (Fig. 8c). The *ARP4* and *ARP6* genes are important for their function in the flowering time phenotype as also highlighted by *in silico* analysis. This suggests that apical-to-floral meristem signalling cascade is controlled by the interaction of up-regulated profilin gene with the coordinated regulation of *ARP4* and *ARP6* genes. This data provided a mechanistic link between *GhPRF1* gene expression and induction of flowering genes’ expression via *ARP4* and *ARP6* genes and provides an insight into the functional characterization of *GhPRF1* gene acting upstream to the flowering mechanism. This observation was further strengthened by the analysis of *Pf*-Si23 line which showed *ARP4* and *ARP6* expression patterns and flowering time similar to control plants.

**Table 2:** Actin-related proteins (ARPs) identified from *Arabidopsis*, their functions and mutant phenotypes

Name	Localization	Function	Mutant Phenotype	Reference
*At*ARP2/*At*ARP3	Nucleus, Cytoplasm, organelle surfaces	Formation of arp2/3 complex; Actin cytoskeleton remodelling; Leaf cell morphogenesis	Development defects in cell shape; Random trichome expansion; Sinuous root hairs	[100, 101]
*At*ARP4	Nucleoplasm	Delayed flowering; Modulation of chromatin structure	Early flowering	[90]
*At*ARP5	Nucleoplasm	Epigenetic control of development; DNA repair	Dwarf plants	[102]
*At*ARP6	Nuclear periphery	Repress flowering	Early-flowering time phenotype	[91]
*At*ARP7	Nucleus	Embryogenesis and plant development	Defects in plant architecture; Plant dwarfism; Small rosette leaves; Embryo lethality	[90]

### Overexpression of *GhPRF1* promotes early flowering without yield penalty

*GhPRF1* was overexpressed in Xanthi to evaluate the function of this gene influencing different physiological and metabolic processes. Upon vegetative growth up to 99-100 dpt, transgenic *Pf*-Ox4 and *Pf*-Ox17 lines showed most apparent flowering time phenotypes exhibiting early flower induction compared to flowering time in the control plants after 112 dpt. (Fig. 6a). Remarkably, overexpression of *GhPRF1* resulted into early flowering time phenotype in tobacco. The flowering time of transgenic lines *Pf*-Ox4 and *Pf*-Ox17 was advanced up to 10–12 days than the untransformed control plants. The overexpression of *GhPRF1* gene led to an increase in the flower number per plant of *Pf*-Ox4 and *Pf*-Ox17 lines compared to control plants (Fig. 6b). Such early flowering phenotype with a significant increase in flower number per plant provided obvious clues for up-regulation of genes involved in possible flowering pathways and processes. This observation was further strengthened by the coordinated expression patterns of certain negative and positive regulators involved in the flowering stimulations. Therefore, overexpression of *GhPRF1* certainly led to early flowering by the up-regulation of the coordinated expression cascade during flower induction.

Due to a significant increase in the number of flowers in *Pf*-Ox4 and *Pf*-Ox17 lines than the control plants, we further investigated whether overexpression of profilin had any adverse effect on floral morphology/anatomy or on pollen formation and pollen viability.

#### Floral development

**Fig. 9:**
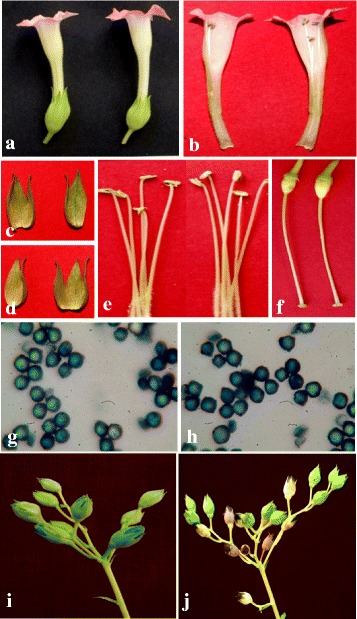
Comparative morphological characters are shown in control flowers and *Pf-*Ox4 overexpression line. **a** Complete flower of control and *Pf-*Ox4 line. **b** Longitudinal section of both control and *Pf-*Ox4 line. **c** & **d** Calyx of control and *Pf-*Ox4 line, respectively. **e** Androecia of control and *Pf-*Ox4 line showing filament and anthers. **f** Gynoecia of control and *Pf-*Ox4 line. **g** Pollen of control line stained with aniline blue. **h** Pollen of *Pf-*Ox4 line stained with aniline blue. **i** & **j** Maturing fruit and their number in control and *Pf-*Ox4 line, respectively

**Fig. 10:**
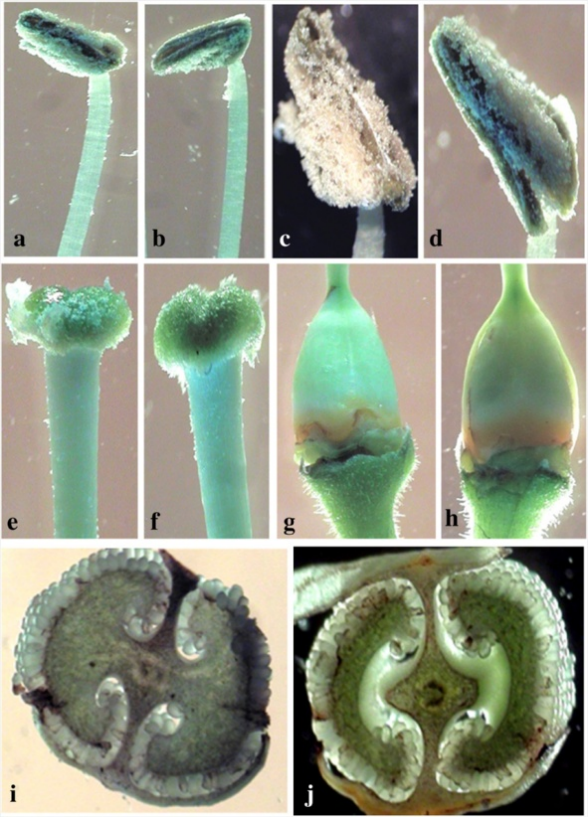
Comparative anatomical features shown in control flowers and *Pf-*Ox4 overexpression line. **a** & **b** Microscopic view of stamen of control and *Pf-*Ox4 line. **c** & **b** Anther of both control and *Pf-*Ox4 line. **e** & **f** Stigma of control and *Pf-*Ox4 line, respectively. **g** & **h** Ovary of control and *Pf-*Ox4 line. **i** & **j** T.S. of a mature ovary of control and *Pf-*Ox4 line, respectively

Flowers of the overexpression *Pf*-Ox4 and *Pf*-Ox17 lines and control plants were analyzed for their morphological features at the initiation and developmental stages. The average flower length varied little between transgenic lines and control plants measuring 53.4 mm and 52.7 mm, respectively (Fig. 9a). Exterior floral parts such as corolla and calyx exhibited minimal differences between transgenic lines and control plants measuring up to 19 mm/15 mm and 50.78 mm/51.3 mm, respectively (Fig. 9b, 9c, 9d; Additional file 1). Among interior floral parts, stamens were present in 4 + 1 orientation and showed no variation in their respective lengths (Fig. 9e). The average height of gynoecium was measured 46.35 mm in transgenic lines whereas 46.31 mm in control plants (Fig. 9f; Additional file 1). Other parameters such as ovary diameter, style length, stigma shape/colour and number of ovules were quantified but no significant variations were observed between transgenic lines and control plants (Additional file 2). Further, to analyze if overexpression *Pf*-Ox4 and *Pf*-Ox17 lines show any anatomical alterations during floral development, the magnitude of organ measurements and cross-sections were compared by microscopy. No significant changes could be observed between transgenic lines and control plants (Fig. 10). Also, no differences in the anthesis-period were recorded between transgenic lines and control plants. This data suggested that overexpression of *GhPRF1* in tobacco does not influence floral organ development.

#### Pollen fertility

To ensure if the overexpression of *GhPRF1* had not influenced the pollen physiology and viability, aniline blue test of pollen grains of *Pf*-Ox4 and *Pf*-Ox17 lines was performed [68]. Generally, pollen grains are considered viable if they had absorbed aniline blue after incubation at room temperature. One-way ANOVA test of pollen grains of both *Pf*-Ox4 and *Pf*-Ox17 lines showed the mean value of staining results equivalent to the pollen grains of control plants (*p* < 0.05) (Fig. 9g, 9h). Data suggest that overexpression of profilin in tobacco does not influence the pollen development and their viability. In result, healthy fruits were observed on both *Pf*-Ox4 and *Pf*-Ox17 transgenic lines without any yield penalty. The external morphology of maturing fruit of these lines along with the number of seeds per fruit was similar to the control plant. However, the number of maturing fruits per plant was higher in *Pf*-Ox4 and *Pf*-Ox17 lines than control plants (Fig. 9i, 9j).

### Overexpression of *GhPRF1* promotes plant height and leaf lamina expansion

It was examined that changes in profilin expression level in transgenic plants produced increased plant height via elongation of internodal regions (Additional file 3). The plant height was recorded up to 53–54 in. in the overexpression *Pf*-Ox4 and *Pf*-Ox17 lines than an average of 38.5 in. in the control plants (Fig. 2e). An average number of leaves in both transgenic lines and control plants were similar counting 34 and 32, respectively. This data indicate that the significant increase in the trait of plant height among transgenic plants was due to increased internode length than the number of nodes produced per plant. It was noted that elongation of internodal regions of both the transgenic *Pf*-Ox4 and *Pf*-Ox17 lines was not consistent across nodes, and the elongation was recorded utmost between 13^th^ and 14^th^ nodes compared to other nodal regions (Additional file 3). The leaf lamina of the two transgenic lines was more expanded in its length and width than to the control plants, but without any changes in the leaf thickness (Additional file 4). Comparative anatomical studies were performed to examine if the expanded leaf lamina of the overexpression *Pf*-Ox4 and *Pf*-Ox17 lines was a result of enhanced cell division, or cellular expansion during leaf growth. Transverse sections of leaves were prepared from transgenic *Pf*-Ox4 and *Pf*-Ox17 lines and control plants. But no radical change in the cell number was recorded in the epidermal layer or parenchymatous tissues. This indicates that expanded leaf lamina is not a result of enhanced cell division but the increased cellular aspect ratio during leaf development. The latter was confirmed by assessing the trichome density on the abaxial surface of the transgenic leaf and control leaf tissues. In typical microscopic view of leaf margin and the midrib regions, significant decrease in the trichome density was observed on the abaxial surface of the transgenic leaf tissues than the control plants (Additional file 5). Since trichome is an extension of epidermal layer, decrease in the trichome density suggested for epidermal cellular expansion rather than increased cell division during leaf development. Increase in plant height and expansion of leaf lamina in *Pf*-Ox4 and *Pf*-Ox17 lines could be the result of profilin-mediated actin polymerization of cell wall that may have led to the cellular expansion.

## Discussion

### Expression evolution of profilin genes under cotton domestication

Genes and *trans*-factor evolved in response to the changing environmental conditions and stress factors influence selective phenotypes and most often leads to plant speciation [69]. In crop plants, the applied selection pressure has been primarily through human-mediated artificial selection (=domestication) underlying morphological transitions in the wild antecedents of modern cultivars. These includes characters such as crop yield, fruit size, reduced seed dormancy, perennial to annual habit, enhanced apical dominance, photoperiodism, and long spinnable natural fiber [2, 6–9, 12].

The domesticated diploid and allotetraploid species of the genus *Gossypium*, have acquired an economically important trait of having long, spinnable fiber that had been evolved under domestication from the wild short fuzz. So, the modern crop having longer fiber is a cumulative outcome of recurrent selection during domestication and recent breeding exercises. Comparative study of such evolutionarily important characters with a morphologically variable ancestor and descendants provided a deep insight into basic principles of selection [1, 12, 14]. Previously, we explored the domestication driven temporal gene expression changes in the elongating fiber cells of the wild and domesticated allotetraploid cotton species *Gossypium barbadense* (AD_2_) [17]. Comparative expression profiling of fiber cells at three developmental stages was performed using a microarray platform which interrogates more than forty- two thousand unigenes. Global gene expression analysis revealed the dynamicity of extraordinarily complex transcriptome of single tetraploid elongating fiber cells. Several differentially expressed gene families constituting various biological functions have been examined at different developmental time-points and between accessions. Three major class of genes have been identified as *i)* hormone-signalling genes *ii)* antioxidant genes, and *iii)* cell-wall structural genes [17].

The latter has drawn more attention where RNAseq data revealed differential overexpression of important cell wall structural protein family of profilin genes up to 400 folds in diploid and tetraploid domesticated forms than their wild ancestors [19]. If so, does the up-regulation of profilin gene family in multiple species reflect directional selection or a co-ordinated stimulus by other important factors? In this direction, present study emphasizes the novel functions of profilins controlling flowering and plant development beyond their traditional role in cell-wall organization. In the current study, profilin1 (*PRF1*) gene of the genus *Gossypium* was characterized for its function considering the genetic diversity of this gene family among cotton homoeologs (present in co-resident A- and D-genomes in allotetraploids) and other homologous sequences (Additional files 6, 7). The distribution of exons and introns in profilin genes across species (Additional file 8), and their homologous sequence comparisons (Additional file 9) highlighted for their conserved genetic design among plant taxa [19].

### *CLV1* and *WUS* expression in response to profilin overexpression

It is apparent that spatial alteration in *CLV1* kinase gene is associated with plant development. More than 30 % down-regulation in the transcript level of *CLV1* in the floral tissues than the vegetative tissues of both *Pf*-Ox4 and *Pf*-Ox17 overexpression lines was observed. This radical alteration in the expression level of such important kinase gene indicates toward its direct role in controlling the apical meristem organization, differentiation and ultimately to the plant phase change. If so, are receptor-kinase genes including *CLV1* receptor-kinase expressed in tight coordination with structural proteins such as profilins? Would the organization of apical-meristem in crop plants be altered by manipulating the expression patterns of profilins? In this direction, experimental validation of other important genes earlier reported for their role in the meristem organization and differentiation such as *KNOX*, *LFY*, *WUS* and *ABP1* is required to confirm the genetic control and interaction relationships with profilin during plant development.

**Fig. 11:**
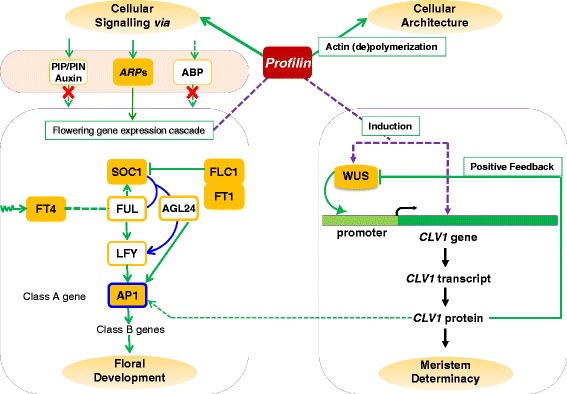
A molecular framework for profilin-mediated activation of apical and reproductive meristem. Different roles of profilin are shown: *(i)* including its classical role in cellular architecture mainly through actin polymerization and depolymerization; cellular signalling mainly through actin-related proteins (*ARP*s). In association with *ARPs*, profilin polymerizes actin, and certain *ARPs* have also been reported for their role in flowering phenomenon. Also, *ARP6* induces *FLC* gene expression leading to the repression of flowering [88, 89]. Such coordinated regulation of flowering time mainly through *ARP* genes with *FLC1* regulator directly influence flower genes expression cascade. *(ii)* its novel roles in apical meristem determinacy via transcriptional activation of *CLV1* gene in the homeodomain *trans*-factor *WUS*- dependent manner; and *(iii)* activation of key flowering regulators for floral development. The latter are known to largely initiate reproductive meristem activation through flowering time controlling genes such as flowering locus T4 (*FT4*) gene which travels from vegetative leaf cells to the initiating floral meristem and in turn up-regulates other flower controlling regulators mainly *SOC1*, *LFY* and ultimately *AP1* which is a class ‘A’ gene and is responsible for the activation of class ‘B’ genes during floral development. Here, we identify important genes whose expression is directly induced by profilin overexpression that furthermore jointly regulate flower primordium initiation. These genes encode known regulators of flower development: *FT4* gene, which specifies the flowering time, *SOC1* transcription factor, which in collaboration with *AGL24* and *LEAFY* (*LFY*) gene up-regulates *AP1* gene, which is a class ‘A’ gene and works as a key regulator of floral development. In parallel, overexpression of profilin down-regulates negative flowering regulators: *FLC1* gene, which suppresses the expression of *SOC1 trans*-factor; and *FT1* gene, which acts as transcriptional inhibitor exclusively in tobacco [67]. Our study reveals a link between profilin and flower primordium initiation mainly *via* up-regulation of *ARP* genes

Yadav et al. [65] have reported that WUS protein is abundant in the neighboring cells of the apical meristem central zone and directly controls the transcriptional activation of *CLV3* through binding to its promoter region. Consecutively, up-regulated *CLV3* gene negatively regulates the WUS protein gradient across meristematic zone which is required for the regulation of stem cell number during floral determinacy. Multiple sequence alignment of *CLV3* gene sequence of *Arabidopsis* with *CLV1* gene sequence of tobacco showed high sequence homology and has shown congruency in their expression patterns. As shown in Fig. 3, the *CLV1* expression level was increased in the vegetative tissues of both *Pf*-Ox4 and *Pf*-Ox17 lines than the reproductive tissues, and conversely, *WUS* expression was reduced in the floral-buds. This reduction in *WUS* expression is considered important for maintaining the low *CLV1* expression level after the formation of floral meristem. It is evident that deletion of *CLV3* promoter region containing WUS-binding sites leads to significant reduction in the promoter activity [70]. Therefore, WUS controls the *CLV1* expression in *GhPRF1* overexpression lines during floral meristem transition, as proposed in Fig. 11. Taken together, this analysis demonstrated that WUS-mediated *CLV1* transcription is maintained in the apical and floral meristems, aiding the early flower initiation in the overexpression lines.

### Overexpression of *GhPRF1* and expression dynamics of key flowering time controlling genes

Profilin genes have been characterized for their contribution to the cell wall organization catalyzing the key step of actin polymerization and depolymerization [71, 72]. Overexpression of profilin in *Arabidopsis* showed longer roots and root hair, broader leaf and accelerating the instigation of flowering [35, 54, 55]. Transient expression of profilin in tobacco cells exhibited extensive cell wall extensions, whereas developmental phases were radically influenced in the cotton fiber cells [57]. A mutated profilin gene in *Arabidopsis* has reduced plant height, leaf size and delayed flowering. In particular, a mutant of *prf1* or *prf2* had defects in rosette leaf morphology and inflorescence architecture, whereas mutant of *PRF3* led to plants with slightly elongated petioles. However, when the mutant plants were complemented with profilin, retention of normal phenotype was observed [59]. By comparing the overexpressed profilin showing elongated roots, increased leaf size and accelerated flowering and subsequent reduction of these traits in *prf*-mutant could reveal valuable information about profilin functioning. However, little is explicit about their role in the complex genetic network which is required for apical meristem activation. In the present study, ectopic overexpression of *trans*-*GhPRF1* in transgenic *Pf*-Ox4 and *Pf*-Ox17 lines demonstrated the hyperactivation of apical meristem and developmental reprogramming targeting early flowering time phenotype. Conversely, depletion of profilin in RNAi lines led to delayed flowering time phenotype and also in flower number per plant.

Concurrently, several transcription factors [46, 73, 74], auxin binding factors [75], peptides [65, 76] and signalling receptor-like kinases [77] are known for their involvement in meristem identity and maintaining balance between cell proliferation and organ formation at shoot/flower meristems [65] (Table 1). The process of flower initiation and its regulation is governed by a complex genetic network where important flower transition and flowering time controlling genes interact during flower development. These observations show that profilin-mediated expression alterations of key negative and positive flowering regulators occur in a coordinated manner. Hence, the hyperactivation of apical meristem and its early conversion into floral meristem is mainly due to increased expression level of positive regulators such as *AP1*, *SOC1* and *FT4* gene transcription which is a probable outcome of enhanced profilin content in the transgenic overexpression lines. During early onset of flowering, *FT4* gene is required to activate *AP1* expression which promotes other functioning genes required for floral meristem differentiation [46]. It is clear with transcription data that *FT4* gene was up-regulated in the vegetative tissues of the overexpression lines which would be transmitted to shoot apex for its morphological transformation into floral primordium [49]. Apparently, *AP1* gene transcription is similar in the 110 dpt and 100 dpt reproductive tissues of both control and *Pf-*Ox4 lines, respectively (Fig. 7a). But early expression elevation in *AP1* gene, perhaps in response to up-regulated *FT4* gene, resulted into early flowering in the overexpression lines than control plants. This is attributed to *AP1* transcription, without which plants showed delayed flowering in *Arabidopsis* [42]. Besides, GA pathway is also required for the activation of important *trans*-factors such as *SOC1* gene along with the suppression of negative flowering regulator *FLC1* gene [78], which is consistent with the expression data.

Therefore, with the over-expression of profilin gene in *Pf*-Ox4 and *Pf*-Ox17 lines, the early flowering phenotype is induced by ARPs-mediated modulation of key regulators. However, these genes could only promote flowering transition if a simultaneous expression reduction occurred in the negative regulators [79, 80]. As proposed, *FLC1* and *FT1* genes negatively regulate *SOC1 trans*-factor and *FT4* genes which collaboratively results into early flowering (Fig. 11). The *FT*-family members are the positive flowering regulators [49, 81], however, *FT1* member of tobacco has been shown to down-regulate key flower controlling genes such as *SOC1* and others [67].

Apart from flowering time modulation, leaf size was also increased in the overexpression *Pf*-Ox4 and *Pf*-Ox17 lines. The size of leaves is firmly controlled by ecological and genetic factors controlling cell expansion and cell division mechanism in a spatial and temporal manner [82]. The increased leaf dimensions observed in the overexpression lines suggested that profilin up-regulation may have influenced the intermediary genetic switches such as *AVP1*, *GRF5*, *JAW*, *BRI1*, and *GA20OX1* genes [83]. Whereas, overexpression of *APC10*, led to increased rate of the cell cycle and produced bigger leaves [84]. Overexpression of *ARGOS*, the homologous protein of *ORGAN SIZE RELATED PROTEIN1* increased the leaf size in plants by elongating proliferative phase of development [85]. However, no report supported the interaction of these genes with temporal expression of profilin protein in the cell. Therefore, it will be of interest to perform the experimental validation of such genes responsible for regulating the leaf dimensions. In the current study, trichome density on the leaf surface and mid- rib of *Pf*-Ox4 and *Pf*-Ox17 lines highlighted for an extra elongation of epidermal cells contributing to the increased leaf size. Hence, increased leaf size and biomass has commercial aspects for several crop plants where leaves are the major source of human usages, such as tobacco, spinaches, cauliflower and more.

In the present study, the overexpression of *GhPRF1* altered the expression of candidate flowering genes, indicating for its role in the intricate mechanism of flower development. However, it is still unclear how profilin regulates the expression of flowering time controlling genes in a coordinated manner? As noted elsewhere, profilin directs several signalling cascades via *PIP* and *PIN* genes which affect auxin concentration and its downstream signalling through polar transport of auxin during floral development [86]. It is also clear that polar auxin transport and downstream signalling is essential for instigation of floral primordia and floral development [42, 87].

### Profilin-mediated regulation of *ARP*s upstream to the flowering gene expression cascade

Flowering process in plants is a complex mechanism and profilin seems to influence factors involved in floral development. So its impact on expression alteration of key flowering genes might also be controlled by few unknown intermediary components. These intermediary molecules/pathways downstream to profilin act either independently or in an overlapping fashion. For example- *i)* AUXIN BINDING PROTEIN1 (ABP1) that acts as binding site for different auxin responses during cellular expansion, cell cycle and cytoskeletal rearrangements, *ii) PIP* signalling genes controlling vesicle trafficking and membrane-cytoskeleton dynamics, channel protein behaviour and signal transduction, and *iii)* Actin-Related Proteins (ARPs) which in proximate association with profilins control actin-nucleation and involved in flower induction and development, transcriptional re-programming at cellular level and cytoskeletal processes.

A parallel outlook of profilin function towards flowering is through *ARPs,* as there are reports suggesting their role in flower development [88, 89]. The *ARPs* act as epigenetic regulator through chromatin remodelling and promotes histone biosynthesis and modification, hence promoting *FLC* transcription [88]. Different plant *ARP*s with their diverse functions as mentioned in Table 2, were considered for the characterization of their role in the observed flowering phenotype of profilin overexpression lines. Interestingly, *ARP4* and *ARP6* were down-regulated in the floral tissues than the vegetative tissues. These *ARP*s have been reported for their direct role in the regulation of flower development especially in defining flowering time phenotype. It has been shown that *ARP4* gene regulates flower development by the modulation of chromatin structure, as the silencing of *ARP4* gene led to early flowering in *Arabidopsis* [90]. It is also evident that profilin polymerizes actin in association with *ARP* genes*.* Different *ARPs* have their roles in flowering phenomenon, for example*, ARP6* induces *FLC* expression and its accumulation led to repression of flowering [88, 89]. Such coordinated regulation of flowering time has been characterized based on the molecular interaction of *ARP4* and *ARP6* genes with negative flowering regulator *FLC1* gene. The *FLC1* gene is positively regulated by *ARP4* and *ARP6* genes, that in result modulate flowering genes’ expression cascade. Among known *ARP*s, *ARP4* and *ARP6* are important for their role in flowering time phenotype as the % silencing of both these genes led to early flowering and flowering time phenotype in proportion [90, 91]. This information was further bolstered by the observation that *ARP4* and *ARP6* silencing lines exhibited a radical reduction in *FLC1* gene transcription levels and uphold their hierarchy in the flowering mechanism [91]. Evidently, *ARP4* and *ARP6* have direct control over vegetative to floral transition during the inception of flowering. Down-expression of both *ARP4* and *ARP6* genes’ transcription in profilin overexpression lines highlighted a mechanistic link to the functional aspects of profilins upstream to the apical-to-floral meristematic gene expression cascade. The present study shows the down-regulation of *ARP* genes in response to overexpressed profilin during developmental phase-change of plants. These findings suggest that profilin is the upstream regulator of *ARPs* in cellular milieu during floral induction (Fig. 11).

## Conclusions

The present study implicates profilin responsive gene network as being involved in the progression of an early flowering phenotype. We provide clues here into flower initiation and developmental genes that may have been up-regulated directly by profilin or via other intermediates in profilin over- and down-expression transgenic lines. Notably, the up-regulation of meristem determinacy *CLV1* gene and its regulatory *WUS* homeodomain *trans*-factor are enhanced in the vegetative tissue (apical meristem), as a result of the conversion of apical-to-floral meristematic tissue. This suggestion that expression up-regulation of key meristem determinacy floral induction genes through *ARP*s controlling flowering time and flower number, was primarily concomitant with the profilin-mediated metabolic transformation of the meristematic cell. This information is further bolstered by the remarkable observation that both independent profilin over-expression and RNAi transgenic stocks had conversely influenced these traits. These observations are mostly veritable at phenotypic, or perhaps at metabolic level highlighting their genesis to be congruent with developmental expression re-arrangements. An exciting prospect for future work will be to dissect the physiological dissimilarities generated by the interacting constituent genes into profilin overexpression and RNAi lines, and to learn about their altered regulation or expression. It would also be interesting to investigate if comparable expression patterns of flower controlling genetic network required for meristem conversion and further development are accompanied in other crops and for other traits, for example, enhanced plant vigor and resistance or tolerance to stress conditions.

## Methods

### Maintenance and generation advancement of tobacco plants

Seeds of tobacco (*Nicotiana tabacum* L.) cultivar Xanthai were sown in 1:1 mixture of soil : soilrite. The germinated healthy seedlings of individual plantlets were grown in sterilized soil mix in the University Green House at 32 ± 1 °C with 16 h light and 8 h dark photoperiodic conditions. Flowers were bagged and tagged for generation advancement in the green house after 110-115 dpt. Along with seeds of Xanthi were grown in vitro by surface sterilization and germination on MS medium [62] at 28 °C ±1 °C with 16 h light and 8 h dark photoperiodic conditions. The control plants were maintained in vitro by regular sub-culturing of nodal explants on the MS medium. These control plants were subsequently transferred to the 1:1 mixture of soil : soilrite for hardening and growth.

### Gene construct design for *GhPRF1* overexpression

Full- length *GhPRF1* cDNA was amplified by PCR using oligonucleotide primer set (Additional file 10) and Q5 polymerase (New England Biolabs) from the cotton cotyledonary leaf. In brief, total RNA was isolated from cotton leaf tissues using Qiagen RNeasy plant mini kit as per the manufacturer’s recommendations. Isolated RNA sample was quantitatively and qualitatively assessed by Nanodrop (Thermo Scientific). One microgram of total RNA was used for synthesizing the complementary DNA using QuantiTect reverse transcription kit (Qiagen). Using Q5 DNA polymerase (NEB), PCR amplification was performed with synthesized cDNA as template and primers as mentioned in Additional file 10, following standard thermal conditions. Gel electrophoresis of the PCR reaction was performed and the amplified product of profilin gene (402 bp) was eluted using Qiagen Gel extraction kit. This amplicon was first sequenced and then used to develop binary vector construct. The PCR product was cloned into the *Nco*I and *Bam*HI restriction sites in the *p*PRT100 cloning vector downstream of the CaMV 35S promoter. The complete cassette of 35S: *GhPRF1*:pA was excised out as a *Hin*dIII fragment and cloned into *Hin*dIII restriction site of binary vector *p*PZP200, along with *nos*:*npt*II gene cassette as a plant selection marker. The modified binary vector was electroporated in *Agrobacterium tumefaciens* strain GV3101.

### Gene construct design for *GhPRF1* silencing

Full- length sense and antisense strands of profilin gene consisting of 402 bp coding for 133 amino acid protein were amplified from the cotyledonary leaf of cotton using specific primers having restriction sites optimized for its cloning into *p*HANNIBAL Plasmid [64] by *Xho*I, *Eco*RI and *Xba*I, *Bam*HI sites, respectively. Following this method, *p*HANNIBAL plasmid was developed containing profilin sense and inverted antisense strand sequences flanking intronic region. *35S:PRF-intron-FRP* region from pHANNIBAL plasmid was digested out using *Not*I restriction enzyme and the sticky ends were polished using HF-Polymerase enzyme. Subsequently, this region was ligated to *p*PZP200 binary vector in *Sma*I restriction site and the *p*PZP200*nos:npt*II*:pA::35S-PRF-intron-FRP:pA* gene construct was developed. This *GhPRF1* silencing construct was further electroporated in *Agrobacterium* strain GV3101 by using GenePulsar (BioRad).

### Gene construct design for *GUS* gene

The gene sequence of *β-glucuronidase* (*gus)* gene was cloned into *p*PZP200 binary vector plasmid under the control of CaMV35S promoter, along with *nos*:*npt*II:pA gene cassette as a plant selection marker. The modified binary vector was electroporated in *Agrobacterium tumefaciens* strain GV3101.

### Development of transgenic tobacco with specific gene constructs

Xanthai leaf explants were used for *Agrobacterium*-mediated genetic transformation following the standard protocol [92]. Transformed explants tissues were allowed to undergo in vitro organogenesis on MS medium supplemented with auxin (NAA = 0.1 mg/l), cytokinin (BAP = 1.0 mg/l) and selection marker kanamycin (100 mg/l). Several putative transgenic shoots were harvested and inoculated on hormone-free MS medium in the test-tubes supplemented with kanamycin (100 mg/l) for at least three successive sub-culturing in vitro. After 30–35 days of shoot growth in vitro, the independent putative transgenic lines were transferred to soil in the green house for hardening and plants were maintained for their appropriate growth and development under controlled conditions.

Genomic DNAs of putative transgenic lines were extracted from leaf tissues using DNeasy DNA isolation kit (Qiagen). Using *npt*II gene-specific primers (Additional file 10), PCR was employed to screen the putative transformants carrying *nos*:*npt*II and 35S:*GhPRF1* gene cassettes. Confirmed transgenic shoots having *GhPRF1* transgene were grown for at least three rounds of sub-culturing on selection medium (kan 50 mg/l). Plantlets grown on selection media were successfully hardened in the green house and considered for expression analyses. PCR analysis for the expression pattern of the *GhPRF1* gene was performed in 23 transgenic plants. The two lines showing high *GhPRF1* expression (*Pf-*Ox4 and *Pf-*Ox17) and one RNAi line (*Pf-*Si23) were selected for analysis.

### Expression analyses of transgenic lines

PCR positive transgenic lines were considered for the analysis of profilin expression level through RT-PCR. Total RNA was extracted from leaf and flower-bud tissues of 4-week old Xanthi wild-type seedlings using RNeasy plant kit (Qiagen) according to the manufacturer’s protocol. The RNA samples having a concentration of at least 1.0 μg were reverse transcribed using QuantiTect Reverse Transcription Kit (Qiagen) according to the manufacturer’s protocol. The replicated RT-PCR was performed using profilin gene-specific primers (Additional file 10) in the leaf and flower tissues of wild type Xanthi plant and PCR positive 35S:*GhPRF1* overexpression lines. The amplified products were subsequently electrophoresed on 1 % agarose gel and observed under UV illumination for relative quantification of transcripts. Normalization of quantitative gene expression data was performed by using previously optimized *GAPDH* and *L25* genes as an internal reference gene for different tobacco tissues [93, 94].

### Morphological analysis of transgenic lines

Transgenic lines confirmed for transgene integration were established in the green house. At regular intervals during vegetative and reproductive phases of plant growth, independent transgenic lines along with control plants were measured for total plant height, internode length, internode number, leaf sheath and leaf lamina length/width, number of flower, size of flower, flowering time, trichome patterning, trichome density and biomass yield. The anatomical features were examined through transverse sections of several vegetative and floral tissues including leaf, stem, petiole, bracts, petals and ovary using Olympus SZ61 microscope.

### Aniline blue staining test of pollen grains

The pollen viability of overexpression, down-expression and control plants was assessed by an aniline blue test. In brief, the mature pollen grains were harvest in the morning at least after 2–3 h of anthesis in at least three biological replicates of each transgenic or control plant. The pollen grains were placed on a glass slide and immersed into diluted aniline blue solution (aniline:water = 1:1). The pollen grains were analysed under Olympus SZ61 microscope and counted for the viable and non-viable pollen grains per optical view of different biological replicates.

## Ethics approval and consent to participate

Not applicable.

## Consent for publication

Not applicable.

## Availability of data and materials

All the data and materials supporting our research findings are contained in the methods section of the manuscript. Also, details are provided in the supplementary data attached with the manuscript.

## Additional files

Additional file 1: Floral dimensions in transgenic *Pf-OX4* line. (PPT 123 kb) Additional file 2: Statistics of flower phenotype of *Pf-Ox4* transgenic lines in comparison to control plant. (PPT 184 kb) Additional file 3 Comparative analysis of internode length at 13^th^ and 20^th^ node of Pf-Ox4 and control plant showing differences at 13^th^ -14^th^ internode. (PPT 150 kb) Additional file 4: Comparative analysis of vegetative tissues showed increased leaf dimensions in *Pf*-Ox4 transgenic plant in comparison to control tobacco plant. (A) Leaf dimensions of 13^th^ leaf of *Pf-Ox4* and control. (B) Leaf dimensions of 20^th^ leaf of *Pf-Ox4* and control plant. (PPT 160 kb) Additional file 5: Trichome density at the leaf margin and midrib of overexpression line (13^th^ leaf from top). (A) Trichome density on mid-rib of *Pf-Ox4*; (B) Trichome density on mid-rib of control leaf; (C) Trichome density on the margin of *Pf-Ox4* leaf; (D) Trichome density on the margin of control leaf. (PPT 2485 kb) Additional file 6: Multiple sequence alignment of six cotton profilin genes showing high homology among the genes. (PDF 150 kb) Additional file 7: Nucleotide sequence analysis of six profilins in cotton showing occurrence of different nucleotides and their percentage frequencies. (PPT 154 kb) Additional file 8: Gene structure of *Nt* *PRF*, *At* *PRF* and *Gh* *PRF* showing three exonic regions intercalated by intron sequence (Gene structure display server, Ver 2). (PPT 136 kb) Additional file 9: Pairwise sequence alignment of tobacco and cotton native profilin protein using Blosum 62 algorithm with gap and extended penalty 10 and 0.5 respectively showing 88.6 % similarity with score value 589.5. (PPT 177 kb) Additional file 10: Primer sequences used in the study. (PPT 207 kb)

## Abbreviations

BAP: 6-Benzylaminopurine
bp: base pair
cm: centimetre
cv.: cultivar
dpt: days post transplantation
hrs: hours
kan: kanamycin
mg/l: milligrams per liter
Mi: minimal
mm: millimetre
MS: Murashige and Skoog
Mx: maximum
NAA: 1-Naphthaleneacetic acid
O: optimal
RT: reproductive tissue
T.S.: transverse section
VT: vegetative tissue
μg/l: micrograms per liter

## References

[1] Li C, Zhou A, Sang T (2006). Rice domestication by reducing shattering. Science.

[2] Wan JM, Jiang L, Tang JY, Wang CM, Hou MY, Jing W, Zhang LX (2005). Genetic dissection of the seed dormancy trait in cultivated rice (*Oryza sativa* L.). Plant Sci.

[3] Doebley J, Stec A, Hubbard L (1997). The evolution of apical dominance in maize. Nature.

[4] Blumler M (1991). Modelling the origins of legume domestication and cultivation. Economic Bot.

[5] Plitman U, Kislev M, Stirton C, Zarucchi J (1989). Reproductive changes induced by domestication. Advances in legume biology.

[6] Applequist WL, Cronn R, Wendel JF (2001). Comparative development of fiber in wild and cultivated cotton. Evol Devel.

[7] Jiang C, Wright R, El-Zik K, Paterson A (1998). Polyploid formation created unique avenues for response to selection in *Gossypium* (cotton). Proc Natl Acad Sci USA.

[8] Smith CW, Cothren JT: Cotton: Origin, History, Technology, and Production. John Wiley & Sons, Inc., New York 1999.

[9] MacArthur JW, Butler L (1938). Size inheritance and geometric growth processes in the tomato fruit. Genetics.

[10] Ugent D (1970). The Potato. Science.

[11] Lu L, Yan W, Xue W, Shao D, Xing Y (2012). Evolution and association analysis of *Ghd7* in rice. PLoS ONE.

[12] Doebley J (2004). The genetics of maize evolution. Ann Rev Genet.

[13] Wang R, Stec A, Hey J, Lukens L, Doebley J (1999). The limits of selection during maize domestication. Nature.

[14] Komatsuda T, Pourkheirandish M, He C, Azhaguvel P, Kanamori H, Perovic D, Stein N, Graner A, Wicker T, Tagiri A (2007). Six-rowed barley originated from a mutation in a homeodomain-leucine zipper I-class homeobox gene. Proc Natl Acad Sci USA.

[15] Lin Z, Li X, Shannon LM, Yeh C-T, Wang ML (2012). Parallel domestication of the Shattering1 genes in cereals. Nat Genet.

[16] Blackman B, Strasburg J, Raduski A, Michaels S, Rieseberg L (2010). The role of recently derived FT paralogs in sunflower domestication. Curr Biol.

[17] Chaudhary B, Hovav R, Rapp R, Verma N, Udall J, Wendel J (2008). Global analysis of gene expression in cotton fibers from wild and domesticated *Gossypium barbadense*. Evol Devel.

[18] Chaudhary B, Hovav R, Flagel L, Mittler R, Wendel J (2009). Parallel expression evolution of oxidative stress-related genes in fiber from wild and domesticated diploid and polyploid cotton (*Gossypium*). BMC Genomics.

[19] Bao Y, Hu G, Flagel L, Salmon A, Bezanilla M, Paterson A, Wang Z, Wendel J (2011). Parallel up-regulation of the profilin gene family following independent domestication of diploid and allopolyploid cotton (*Gossypium*). Proc Natl Acad Sci USA.

[20] Christensen H, Ramachandran S, Tan C, Surana U, Dong C, Chua N (1996). *Arabidopsis* profilins are functionally similar to yeast profilins: identification of a vascular bundle-specific profilin and a pollen-specific profilin. Plant J.

[21] Kovar DR, Drobak BK, Staiger CJ (2000). Maize profilin isoforms are functionally distinct. Plant Cell.

[22] Staiger CJ, Gibbon BC, Kovar DR, Zonia LE (1997). Profilin and actin depolymerizing factor: Modulators of actin organization in plants. Trends Plant Sci.

[23] Staiger CJ, Goodbody KC, Hussey PJ, Valenta R, Drobak BK, Lloyd CW (1993). The profilin multigene family of maize: differential expression of three isoforms. Plant J.

[24] Huang SR, McDowell JM, Weise MJ, Meagher RB (1996). The *Arabidopsis* profilin gene family. Evidence for an ancient split between constitutive and pollen-specific profilin genes. Plant Physiol.

[25] Kandasamy MK, McKinney EC, Meagher RB (2002). Plant profilin isovariants are distinctly regulated in vegetative and reproductive tissues. Cell Motil Cytoskeleton.

[26] Mittermann I, Heiss S, Kraft D, Valenta R, Heberle-Bors E (1996). Molecular characterization of profilin isoforms from tobacco (*Nicotiana tabacum*) pollen. Sexual Plant Reprod.

[27] Schütz I, Gus-Mayer S, Schmelzer E (2006). Profilin and Rop GTPases are localized at infection sites of plant cells. Protoplasma.

[28] Magdolen V, Drubin DG, Mages G, Bandlow W (1993). High levels of profilin suppress the lethality caused by overproduction of actin in yeast cells. FEBS Lett.

[29] Magdolen V, Oechsner U, Müller G, Bandlow W (1988). The intron-containing gene for yeast profilin (PFY) encodes a vital function. Mol Cell Biol.

[30] Pantaloni D, Carlier M-F (1993). How profilin promotes actin filament assembly in the presence of thymosin β4. Cell.

[31] Baum B, Perrimon N (2001). Spatial control of the actin cytoskeleton in *Drosophila* epithelial cells. Nat Cell Biol.

[32] Verheyen EM, Cooley L (1994). Profilin mutations disrupt multiple actin-dependent processes during *Drosophila* development. Development.

[33] Yu LX, Nasrallah J, Valenta R, Parthasarathy MV (1998). Molecular cloning and mRNA localization of tomato pollen profilin. Plant Mol Bio.

[34] Vidali L, Perez HE, Valdes Lopez V, Noguez R, Zamudio F, Sanchez F (1995). Purification, characterization, and cDNA cloning of profilin from *Phaseolus vulgaris*. Plant Physiol.

[35] Ramachandran S, Christensen HE, Ishimaru Y, Dong CH, Chao-Ming W, Cleary AL, Chua NH (2000). Profilin plays a role in cell elongation, cell shape maintenance, and flowering in *Arabidopsis*. Plant Physiol.

[36] Carlsson L, Nystrom LE, Sundkvist I, Markey F, Lindberg U (1977). Actin polymerizability is influenced by profilin, a low molecular weight protein in non-muscle cells. J Mol Biol.

[37] Schlüter K, Jockusch BM, Rothkegel M (1997). Profilins as regulators of actin dynamics. Biochim Biophys Acta.

[38] Mullins RD, Heuser JA, Pollard TD (1998). The interaction of Arp2/3 complex with actin:nucleation, high affinity pointed end capping, and formation of branching networks of filaments. ProcNatl Acad Sci USA.

[39] Alvarez-Martinez MT, Mani JC, Porte F, Faivre-Sarrailh C, Liautard JP, Sri Widada J (1996). Characterization of the interaction between annexin I and profilin. Eur J Biochem.

[40] Mahoney NM, Rozwarski DA, Fedorov E, Fedorov AA, Almo SC (1999). Profilin binds proline-rich ligands in two distinct amide backbone orientations. Nat Struct Biol.

[41] Sohn RH, Goldschmidt-Clermont PJ (1994). Profilin: At the crossroads of signal transduction and the actin cytoskeleton. BioEssays.

[42] Chen Z, Ye M, Su X, Liao W, Ma H, Gao K, Lei B, An X (2015). Overexpression of AtAP1M3 regulates flowering time and floral development in Arabidopsis and effects key flowering-related genes in poplar. Transgenic Res.

[43] Yant L, Mathieu J, Dinh TT, Ott F, Lanz C, Wollmann H, Chen X, Schmid M (2010). Orchestration of the floral transition and floral development in *Arabidopsis* by the bifunctional transcription factor APETALA2. The Plant Cell.

[44] Wang L, Liang H, Pang J, Zhu M (2004). Regulation Network and Biological Roles of LEAFY in Arabidopsis thaliana in Floral Development. Hereditas.

[45] Immink RGH, Posé D, Ferrario S, Ott F, Kaufmann K, Valentim FL, de Folter S, Van der Wal F, Dijk ADJ, Schmid M (2012). Characterization of SOC1’s central role in flowering by the identification of its upstream and downstream regulators. Plant Physiol.

[46] Lee J, Lee I (2010). Regulation and function of SOC1, a flowering pathway integrator. J Exp Bot.

[47] Laurie RE, Diwadkar P, Jaudal M, Zhang L, Hecht V, Wen J, Tadege M, Mysore KS, Putterill J, Weller JL (2011). The Medicago truncatula FLOWERING LOCUS T homologue, MtFTa1, is a key regulator of flowering time. Plant Physiol.

[48] Guo Y-L, Todesco M, Hagmann J, Das S, Weigel D (2012). Independent FLC mutations as causes of flowering-time variation in *Arabidopsis thaliana* and *Capsella rubella*. Genetics.

[49] Abe M, Kobayashi Y, Yamamoto S, Daimon Y, Yamaguchi A, Ikeda Y, Ichinoki H, Notaguchi M, Goto K, Araki T (2005). FD, a bZIP protein mediating signals from the floral pathway integrator FT at the shoot apex. Science.

[50] Huang T, Böhlenius H, Eriksson S, Parcy F, Nilsson O (2005). The mRNA of the *Arabidopsis* gene FT moves from leaf to shoot apex and induces flowering. Science.

[51] Jeong JH, Song HR, Ko JH, Jeong YM, Kwon YE, Seol JH, Amasino RM, Noh B, Noh YS (2009). Repression of FLOWERING LOCUS T chromatin by functionally redundant histone H3 lysine 4 demethylases in *Arabidopsis*. PLoS One.

[52] Michaels SD, Amasino RM (1999). FLOWERING LOCUS C encodes a novel MADS domain protein that acts as a repressor of fowering. Plant Cell.

[53] Noh B, Lee SH, Kim HJ, Yi G, Shin EA, Lee M, Jung KJ, Doyle MR, Amasino RM, Noh YS (2004). Divergent roles of a pair of homologous Jumonji/Zinc-Finger–class transcription factor proteins in the regulation of *Arabidopsis* flowering time. The Plant Cell.

[54] Chua NH, Ramachandran S, Christensen HEM (2002). Alteration of plant morphology by control of profilin expression. Google Patents.

[55] Haarer B, Lillie S, Adams A, Magdolen V, Bandlow W, Brown SS (1990). Purification of profilin from *Saccharomyces cerevisiae* and analysis of profilin-deficient cells. J Cell Biol.

[56] Wang HY, Yu Y, Chen ZL, Xia GX (2005). Functional characterization of *Gossypium hirsutum* profilin 1 gene (GhPFN1) in tobacco suspension cells. Characterization of *in vivo* functions of a cotton profilin gene. Planta.

[57] Wang J, Wang H, Zhao P, Han L, Jiao G, Zheng Y, Huang S, Xia G (2010). Overexpression of a profilin (GhPFN2) promotes the progression of developmental phases in cotton fibers. Plant Cell Physiol.

[58] McKinney EC, Kandasamy MK, Meagher RB (2001). Small changes in the regulation of one *Arabidopsis* profilin isovariant, PRF1, alter seedling development. Plant Cell.

[59] Müssar KJ, Kandasamy MK, McKinney EC, Meagher RB (2015). *Arabidopsis* plants deficient in constitutive class profilins reveal independent and quantitative genetic effects. BMC Plant Biology.

[60] Taylor-Teeples M, Lin L, de Lucas M, Turco G, Toal TW, Gaudinier A, Young NF, Trabucco GM (2014). An Arabidopsis gene regulatory network for secondary cell wall synthesis. Nature.

[61] Kay R, Chan A, Daly M, McPherson J (1987). Duplication of CaMV 35S promoter sequences creates a strong enhancer for plant genes. Science.

[62] Murashige T, Skoog F (1962). A revised medium for rapid growth and bio-assay with tobacco tissue cultures. Physiol Plant.

[63] Pandey DK, Singh A, Chaudhary B (2012). Boron-mediated plant somatic embryogenesis: A provocative model. J Bot.

[64] Wesley S, Helliwell C, Smith N, Wang M, Rouse D, Liu Q, Gooding P, Singh S, Abbott D, Stoutjesdijk P (2001). Construct design for efficient, effective and high throughput gene silencing in plants. Plant J.

[65] Yadav RK, Perales M, Gruel J, Girke T, Jönsson H, Reddy GV (2011). WUSCHEL protein movement mediates stem cell homeostasis in the *Arabidopsis* shoot apex. Genes Dev.

[66] Szklarczyk D, Franceschini A, Wyder S, Forslund K, Heller D, Huerta-Cepas J, Simonovic M, Roth A, Santos A, Tsafou KP (2015). STRING v10: protein–protein interaction networks, integrated over the tree of life. Nucl Acids Res.

[67] Harig L, Beinecke FA, Oltmanns J, Muth J, Müller O, Rüping B, Twyman RM, Fischer R, Prüfer D, Noll GA (2012). Proteins from the FLOWERING LOCUS T-like subclade of the PEBP family act antagonistically to regulate floral initiation in tobacco. Plant J.

[68] Adhikari KN, Campbell CG (1998). In vitro germination and viability of buckwheat (Fagopyrum esculentum Moench) pollen. Euphytica.

[69] Hoffman AA, Hercus MJ (2000). Environmental stress as an evolutionary force. BioScience.

[70] Muller R, Borghi L, Kwiatkowska D, Laufs P, Simon R (2006). Dynamic and compensatory responses of Arabidopsis shoot and floral meristems to CLV3 signaling. Plant Cell.

[71] Bubb MR, Yarmola EG, Gibson BG, Southwick FS (2003). Depolymerization of actin filaments by profilin effects of profilin on capping protein function. J Biol Chem.

[72] Pring M, Weber A, Bubb MR (1992). Profilin-actin complexes directly elongate actin filaments at the barbed end. Biochemistry.

[73] Weigel D, Alvarez J, Smyth D, Yanofsky M, Meyerowitz E (1992). LEAFY controls floral meristem identity in Arabidopsis. Cell.

[74] Weigel D, Nilsson O (1995). A developmental switch sufficient for flower initiation in diverse plants. Nature.

[75] Xu T, Dai N, Chen J, Nagawa S, Cao M, Li H, Zhou Z, Chen X, De RR, Rakusova H (2014). Cell surface ABP1-TMK auxin-sensing complex activates ROP GTPase signaling. Science.

[76] Trotochaud AE, Hao T, Wu G, Yang Z, Clark SE (1999). The CLAVATA1 receptor-like kinase requires CLAVATA3 for its assembly into a signaling complex that includes KAPP and a Rho-related protein. Plant Cell.

[77] Clark SE, Williams RW, Meyerowitz EM (1997). The CLAVATA1 gene encodes a putative receptor kinase that controls shoot and floral meristem size in *Arabidopsis*. Cell.

[78] Moon J, Suh S-S, Lee H, Choi K-R, Hong CB, Paek N-C, Kim S-G, Lee I (2003). The SOC1 MADS-box gene integrates vernalization and gibberellin signals for flowering in *Arabidopsis*. Plant J.

[79] Amasino RM, Michaels SD (2010). The timing of flowering. Plant Physiol.

[80] Blázquez MA, Soowal LN, Lee I, Weigel D (1997). LEAFY expression and flower initiation in *Arabidopsis*. Dev Camb Engl.

[81] Wigge PA, Kim MC, Jaeger KE, Busch W, Schmid M, Lohmann JU, Weigel D (2005). Integration of spatial and temporal information during floral induction in Arabidopsis. Science.

[82] Walter A, Silk WK, Schurr U (2009). Environmental effects on spatial and temporal patterns of leaf and root growth. Ann Rev Plant Biol.

[83] Gonzalez N, De BS, Sulpice R, Jikumaru Y, Chae E, Dhondt S, Van DT, De ML, Weigel D, Kamiya Y (2010). Increased leaf size: different means to an end. Plant Physiol.

[84] Eloy NB, de Freitas Lima M, Van Damme D, Vanhaeren H, Gonzalez N, De Milde L, Hemerly AS, Beemster GT, Inzé D, Ferreira PC (2011). The APC/C subunit 10 plays an essential role in cell proliferation during leaf development. Plant J.

[85] Feng G, Qin Z, Yan J, Zhang X, Hu Y (2011). *Arabidopsis* ORGAN SIZE RELATED1 regulates organ growth and final organ size in orchestration with ARGOS and ARL. New Phytol.

[86] Mei Y, Jia W, Chu Y, Xue H (2012). *Arabidopsis* phosphatidylinositol monophosphate 5-kinase 2 is involved in root gravitropism through regulation of polar auxin transport by affecting the cycling of PIN proteins. Cell Res.

[87] Aloni R, Aloni E, Langhans M, Ullrich CI (2006). Role of auxin in regulating *Arabidopsis* flower development. Planta.

[88] Deal RB, Kandasamy MK, McKinney EC, Meagher RB (2005). The nuclear actin-related protein ARP6 is a pleiotropic developmental regulator required for the maintenance of FLOWERING LOCUS C expression and repression of flowering in *Arabidopsis*. Plant Cell.

[89] Martin-Trillo M, Lazaro A, Poethig RS, Gomez-Mena C, Pineiro MA, Martinez-Zapater JM, Jarillo JA (2006). EARLY IN SHORT DAYS 1 (ESD1) encodes ACTIN-RELATED PROTEIN 6 (AtARP6), a putative component of chromatin remodelling complexes that positively regulates FLC accumulation in *Arabidopsis*. Development.

[90] Kandasamy MK, Deal RB, McKinney EC, Meagher RB (2005). Silencing the nuclear actin-related protein *At*ARP4 in *Arabidopsis* has multiple effects on plant development, including early flowering and delayed floral senescence. Plant J.

[91] Choi K, Kim S, Kim SY, Kim M, Hyun Y, Lee H, Choe S, Kim S-G, Michaels S, Lee I (2005). SUPPRESSOR OF FRIGIDA3 encodes a nuclear ACTIN-RELATED PROTEIN6 required for floral repression in *Arabidopsis*. The Plant Cell.

[92] Kuluev BR, Knyazev AB, Lebedev YP, Postrigan BN, Chemeris AV (2012). Obtaining transgenic tobacco plants expressing conserved regions of the AINTEGUMENTA gene in antisense orientation. Russian J Plant Physiol.

[93] Pandey DK, Chaudhary B (2014). Oxidative stress responsive SERK1 gene directs the progression of somatic embryogenesis in cotton (*Gossypium hirsutum* L. cv. Coker 310). Am J Plant Sci.

[94] Schmidt GW, Delaney SK (2010). Stable internal reference genes for normalization of real-time RT-PCR in tobacco (*Nicotiana tabacum*) during development and abiotic stress. J Mol Genet Genomics.

[95] Sakamoto T, Kamiya N, Iwahori S, Matsuoka M (2001). KNOX homeodomain protein directly suppresses the expression of a gibberellin biosynthetic gene in the tobacco shoot apical meristem. Genes Dev.

[96] Vollbrecht E, Reiser L, Hake S (2000). Shoot meristem size is dependent on inbred background and presence of the maize homeobox gene, knotted1. Development.

[97] Busch W, Miotk A, Ariel F, Zhao Z, Forner J, Daum G, Suzaki T, Schuster C, Schultheiss S, Leibfried A (2010). Transcriptional control of a plant stem cell niche. Dev Cell.

[98] Pajoro A, Madrigal P, Muino J, Matus J, Jin J, Mecchia M, Debernardi J, Palatnik J, Balazadeh S, Arif M (2014). Dynamics of chromatin accessibility and gene regulation by MADS domain transcription factors in flower development. Genome Biol.

[99] Sassi M, Ali O, Boudon F, Cloarec G, Abad U, Cellier C, Chen X, Gilles B, Milani P, Friml J (2014). An auxin-mediated shift toward growth isotropy promotes organ formation at the shoot meristem in *Arabidopsis*. Curr Biol.

[100] Li S, Blanchoin L, Yang Z, Lord EM (2003). The putative *Arabidopsis* Arp2/3 complex controls leaf cell morphogenesis. Plant Physiol.

[101] Zhang C, Mallery EL, Szymanski D (2013). ARP2/3 localization in *Arabidopsis* leaf pavement cells: a diversity of intracellular pools and cytoskeletal interactions. Frontiers Plant Sci.

[102] Kandasamy MK, McKinney EC, Deal RB, Smith AP, Meagher RB (2009). *Arabidopsis* actin-related protein ARP5 in multicellular development and DNA repair. Dev Biol.

