# Supplementary figures and images for: Domestication-driven Gossypium profilin 1 (GhPRF1) gene transduces early flowering phenotype in tobacco by spatial alteration of apical/floral-meristem related gene expression
Source: BMC Plant Biol. 2016 May 13;16:112. doi: 10.1186/s12870-016-0798-0 (PMC4866011; doi:10.1186/s12870-016-0798-0)

## Slide 1
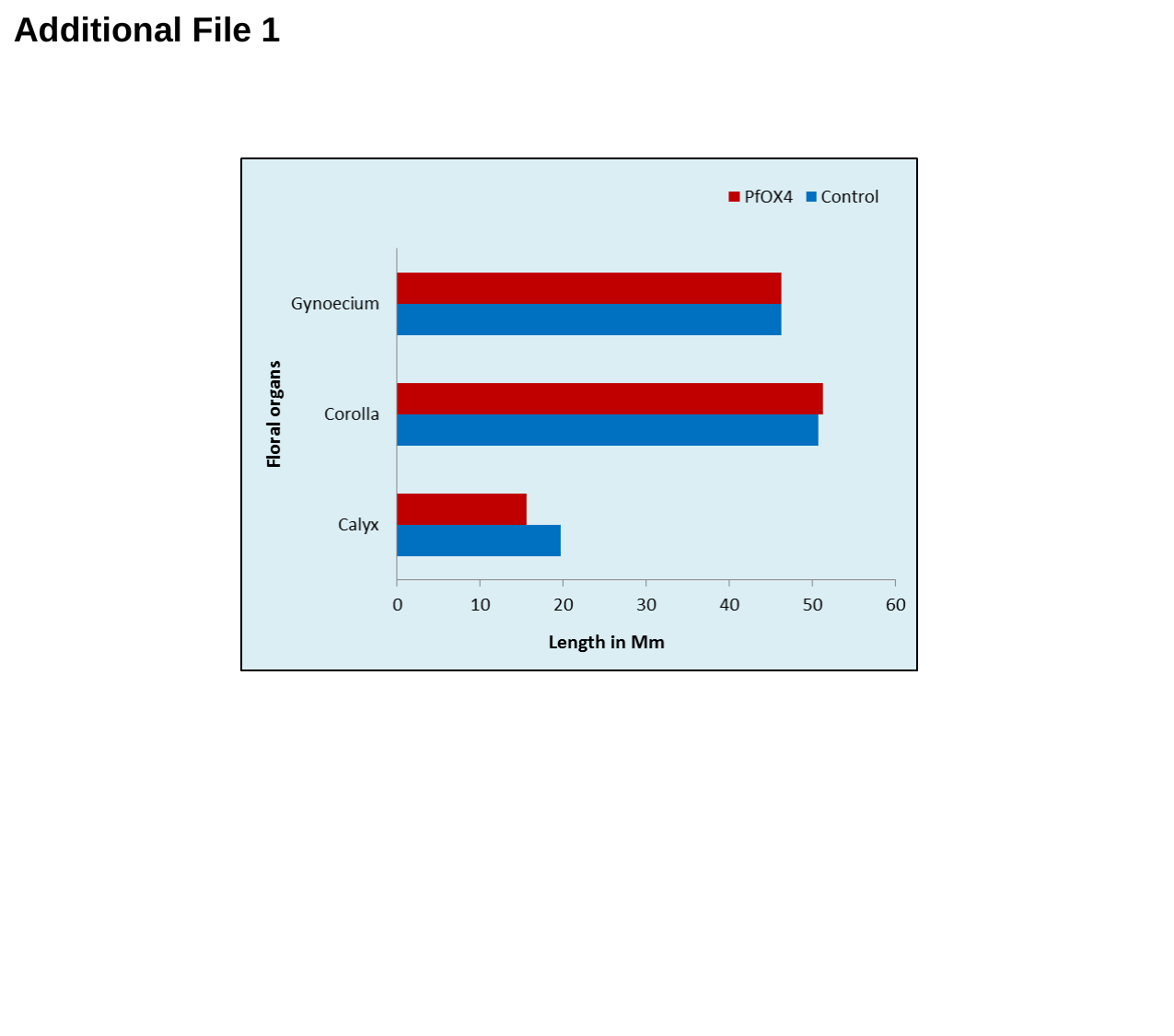

Additional File 1

Supplement: Additional file 1: — Floral dimensions in transgenic Pf-OX4 line. (PPT 123 kb) [file 12870_2016_798_MOESM1_ESM.ppt]

## Slide 1
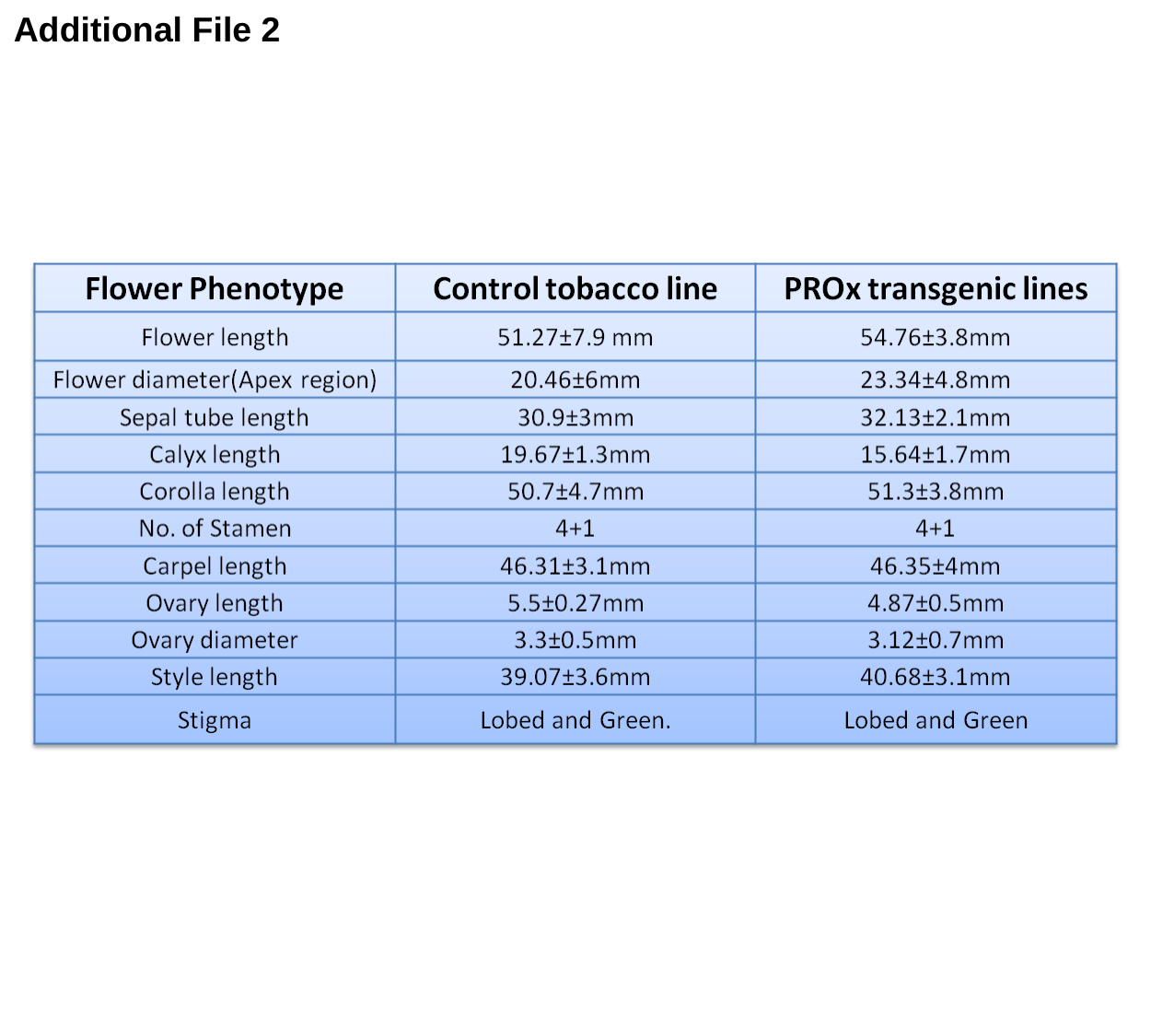

Additional File 2

Supplement: Additional file 2: — Statistics of flower phenotype of Pf-Ox4 transgenic lines in comparison to control plant. (PPT 184 kb) [file 12870_2016_798_MOESM2_ESM.ppt]

## Slide 1
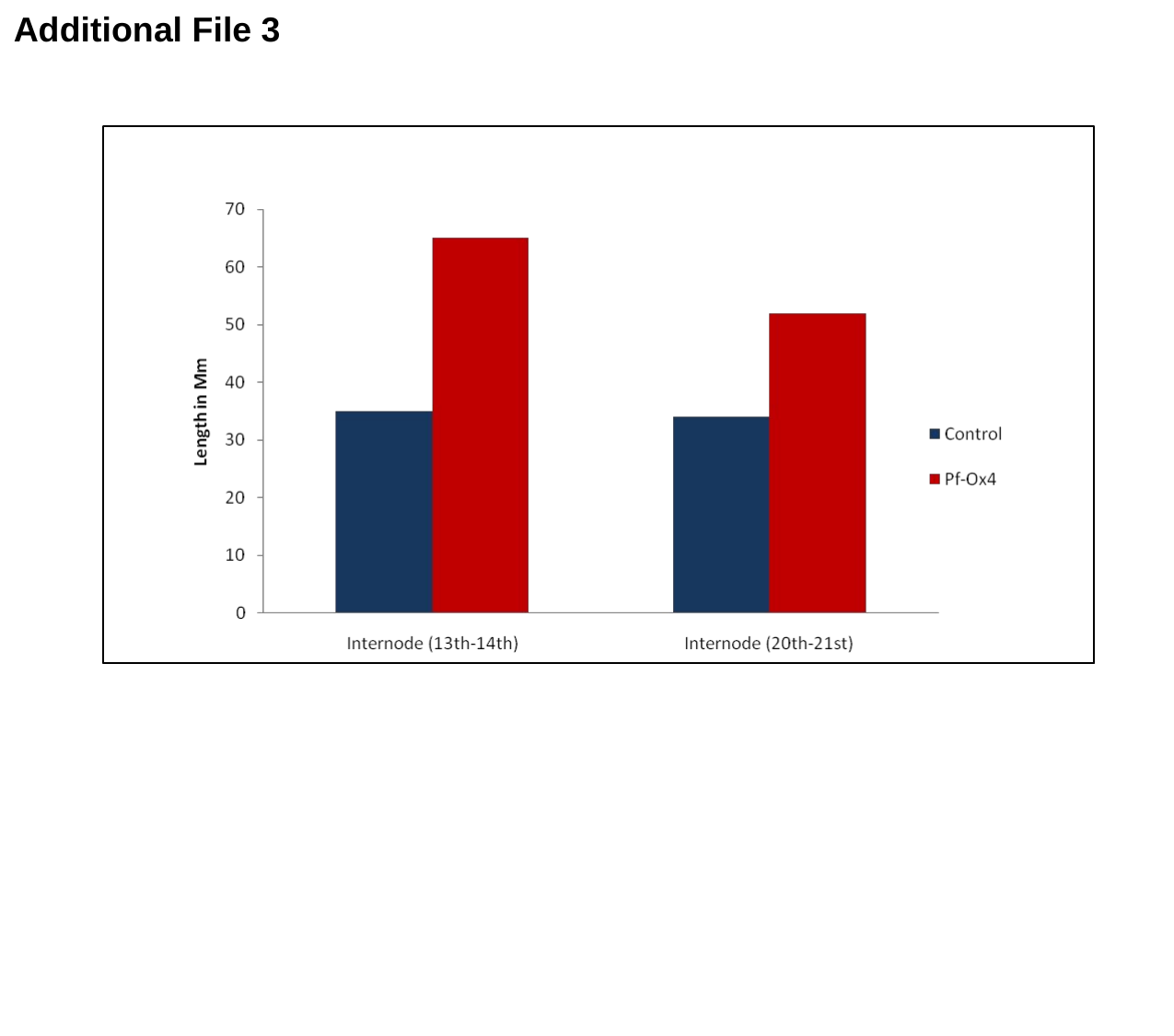

Additional File 3

Supplement: Additional file 3 — Comparative analysis of internode length at 13th and 20th node of Pf-Ox4 and control plant showing differences at 13th -14th internode. (PPT 150 kb) [file 12870_2016_798_MOESM3_ESM.ppt]

## Slide 1
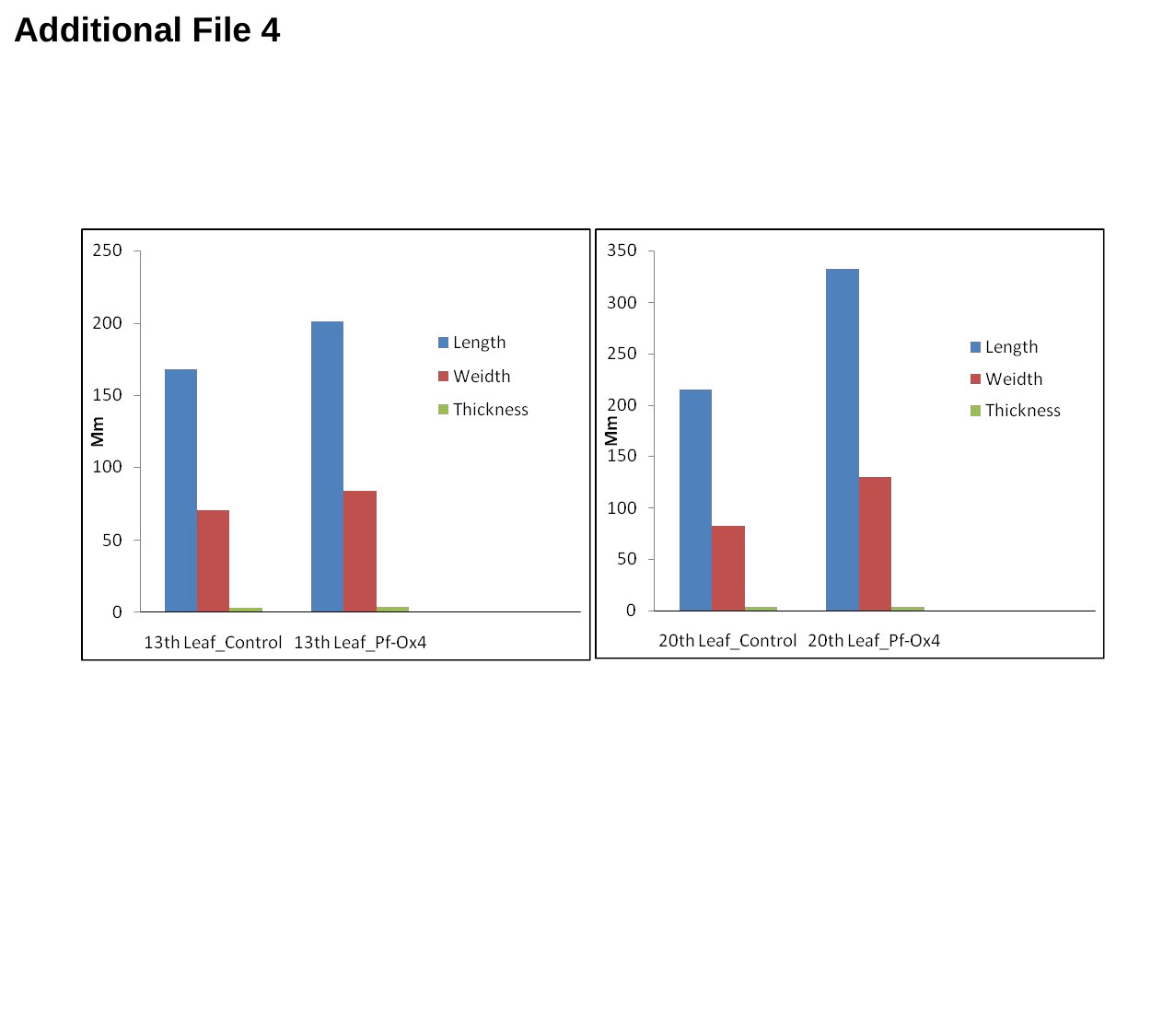

Additional File 4

Supplement: Additional file 4: — Comparative analysis of vegetative tissues showed increased leaf dimensions in Pf-Ox4 transgenic plant in comparison to control tobacco plant. (A) Leaf dimensions of 13th leaf of Pf-Ox4 and control. (B) Leaf dimensions of 20th leaf of Pf-Ox4 and control plant. (PPT 160 kb) [file 12870_2016_798_MOESM4_ESM.ppt]

## Slide 1
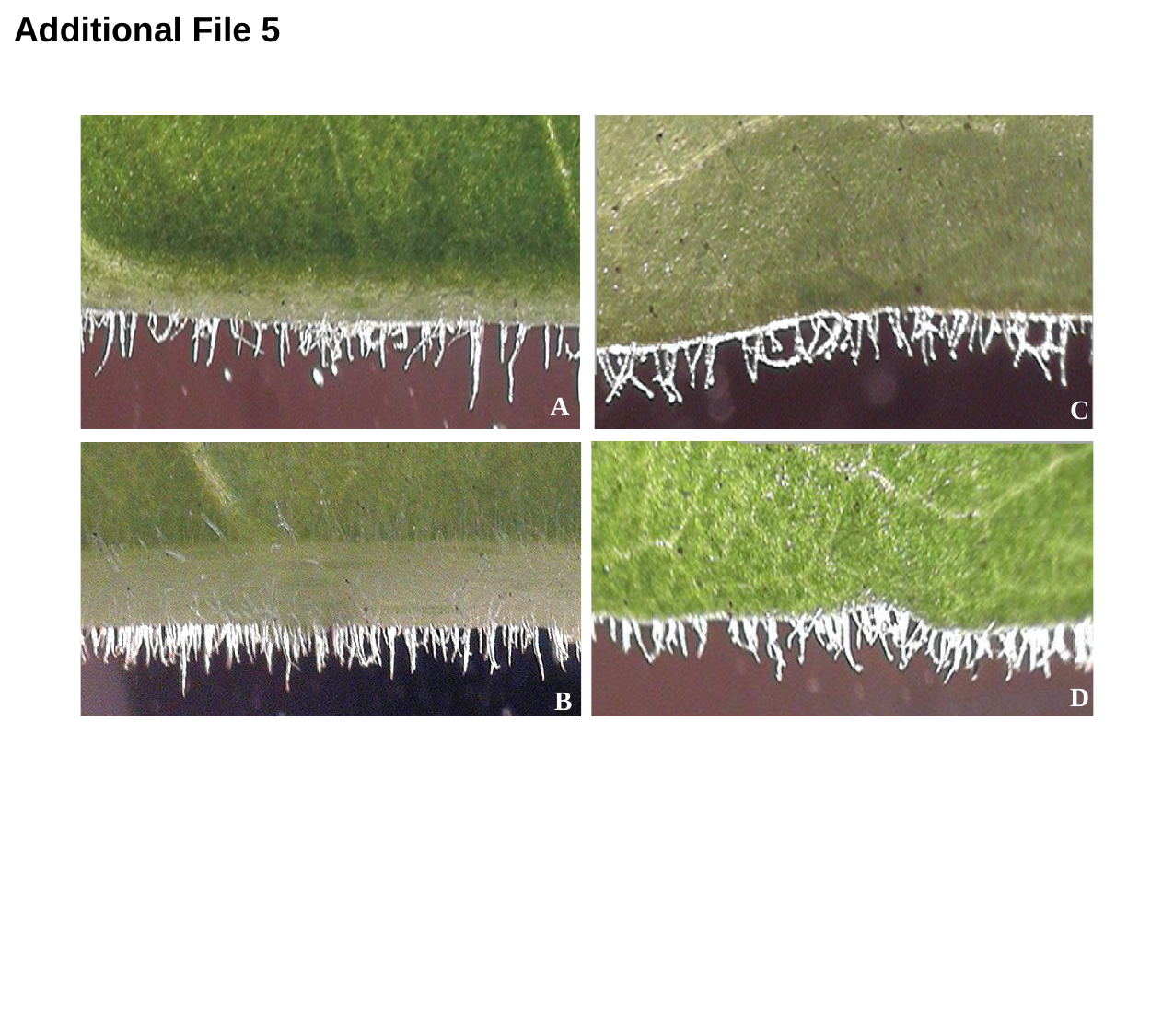

Additional File 5
A
C
D
B

Supplement: Additional file 5: — Trichome density at the leaf margin and midrib of overexpression line (13th leaf from top). (A) Trichome density on mid-rib of Pf-Ox4; (B) Trichome density on mid-rib of control leaf; (C) Trichome density on the margin of Pf-Ox4 leaf; (D) Trichome density on the margin of control leaf. (PPT 2485 kb) [file 12870_2016_798_MOESM5_ESM.ppt]

## Slide 1
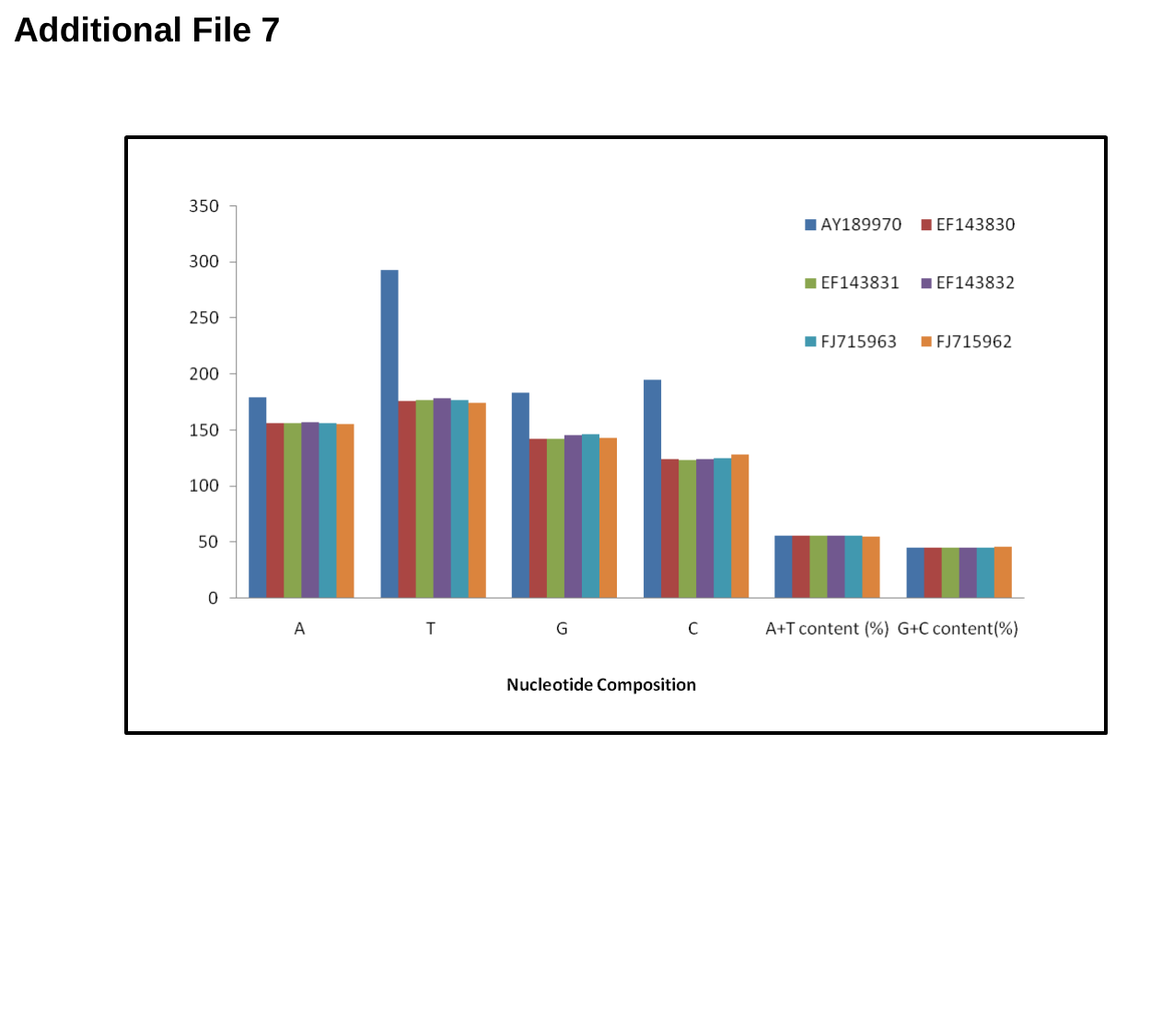

Additional File 7

Supplement: Additional file 7: — Nucleotide sequence analysis of six profilins in cotton showing occurrence of different nucleotides and their percentage frequencies. (PPT 154 kb) [file 12870_2016_798_MOESM7_ESM.ppt]

## Slide 1
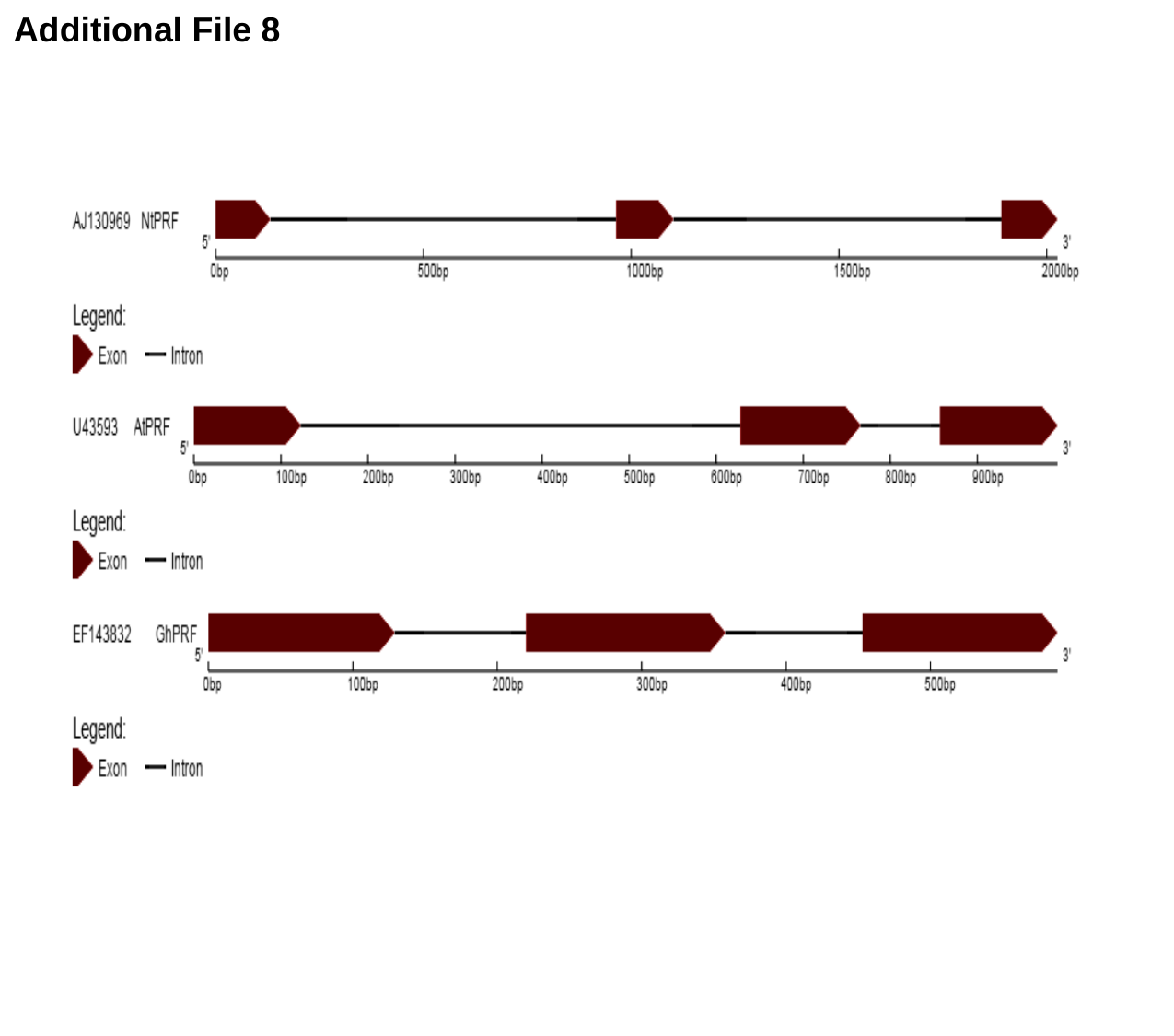

Additional File 8

Supplement: Additional file 8: — Gene structure of Nt PRF, At PRF and Gh PRF showing three exonic regions intercalated by intron sequence (Gene structure display server, Ver 2). (PPT 136 kb) [file 12870_2016_798_MOESM8_ESM.ppt]

## Slide 1
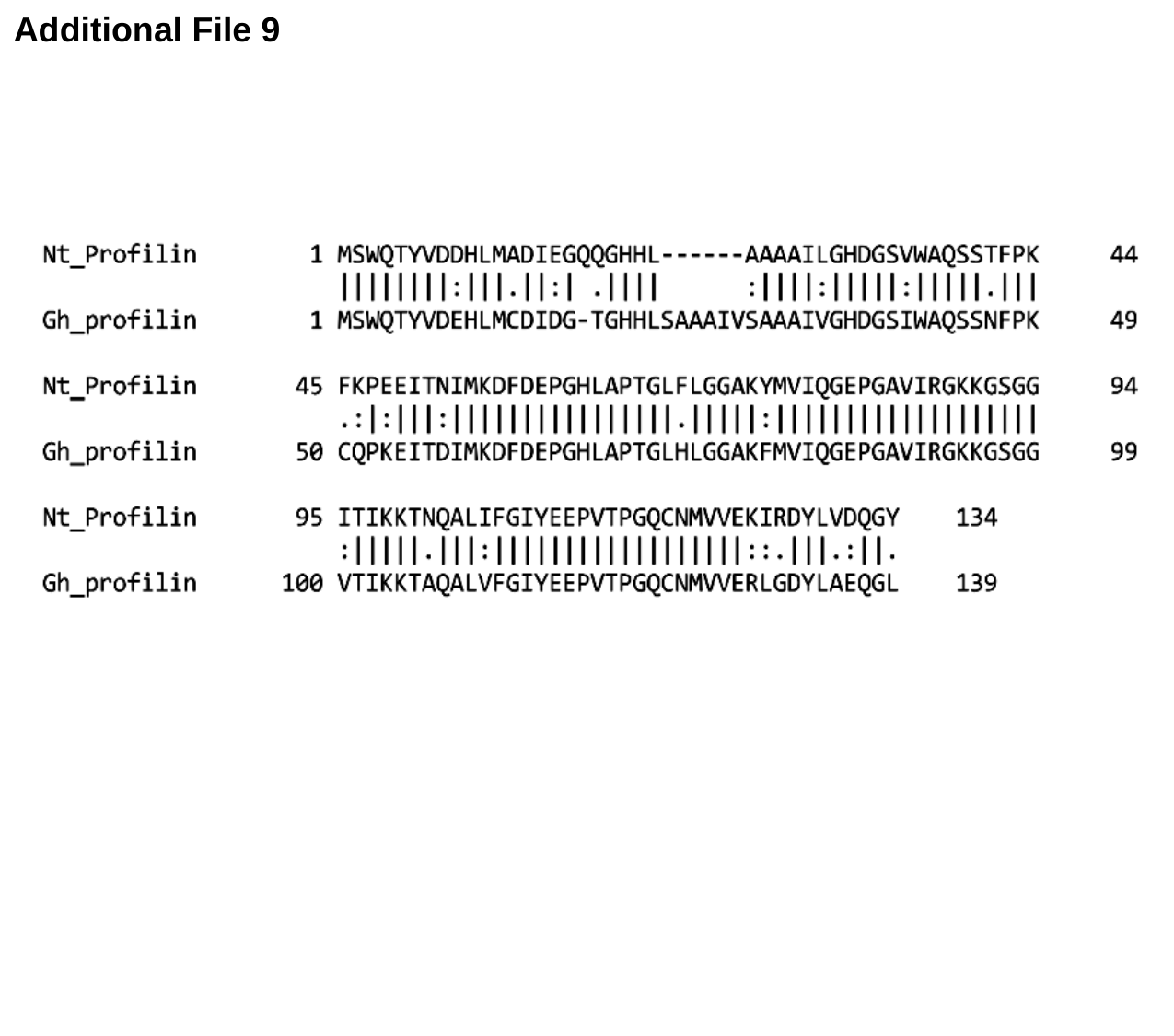

Additional File 9

Supplement: Additional file 9: — Pairwise sequence alignment of tobacco and cotton native profilin protein using Blosum 62 algorithm with gap and extended penalty 10 and 0.5 respectively showing 88.6 % similarity with score value 589.5. (PPT 177 kb) [file 12870_2016_798_MOESM9_ESM.ppt]
