# Supplementary material for: Domestication-driven Gossypium profilin 1 (GhPRF1) gene transduces early flowering phenotype in tobacco by spatial alteration of apical/floral-meristem related gene expression
Source: BMC Plant Biol. 2016 May 13;16:112. doi: 10.1186/s12870-016-0798-0 (PMC4866011; doi:10.1186/s12870-016-0798-0)
Supplement: Additional file 6: — Multiple sequence alignment of six cotton profilin genes showing high homology among the genes. (PDF 150 kb) [file 12870_2016_798_MOESM6_ESM.pdf]

Additional File 6

```
gi|217331645|gb|AY189970.2|_Go  ACCATTACCCTTCTCCCCCTTTCCTCCCATATTCCTCTTTCCCAATACCC
gi|133925930|gb|EF143830.1|_Go  A-------------------------------------------------
gi|133925932|gb|EF143831.1|_Go  A-------------------------------------------------
gi|133925934|gb|EF143832.1|_Go  A-------------------------------------------------
gi|260871354|gb|FJ715963.1|_Go  A-------------------------------------------------
gi|260871352|gb|FJ715962.1|_Go  A-------------------------------------------------

gi|217331645|gb|AY189970.2|_Go  TTTCACCCTTGCTAATAAAACCACCTTATTAACATACCTAACCATGTCGT
                                                                    MetSer
gi|133925930|gb|EF143830.1|_Go  --------------------------------CATACCTAACCATGTCGT
                                                                    MetSer
gi|133925932|gb|EF143831.1|_Go  --------------------------------CATACCTAACCATGTCGT
                                                                    MetSer
gi|133925934|gb|EF143832.1|_Go  --------------------------------CATACCTAACCATGTCGT
                                                                    MetSer
gi|260871354|gb|FJ715963.1|_Go  --------------------------------CATACCTAACCATGTCGT
                                                                    MetSer
gi|260871352|gb|FJ715962.1|_Go  --------------------------------CATACCTAACCATGTCGT
                                                                    MetSer

gi|217331645|gb|AY189970.2|_Go  GGCAAACCTACGTTGATGAGCACTTGATGTGTGACATCGATGGCACTGGT
                               TrpGlnThrTyrValAspGluHisLeuMetCysAspIleAspGlyThrGly
gi|133925930|gb|EF143830.1|_Go  GGCAAACCTACGTTGATGAGCACTTGATGTGTGACATCGATGGCACTGGT
                               TrpGlnThrTyrValAspGluHisLeuMetCysAspIleAspGlyThrGly
gi|133925932|gb|EF143831.1|_Go  GGCAAACCTACGTTGATGAGCACTTGATGTGTGACATCGATGGCACTGGT
                               TrpGlnThrTyrValAspGluHisLeuMetCysAspIleAspGlyThrGly
gi|133925934|gb|EF143832.1|_Go  GGCAAACCTACGTTGATGAGCACTTGATGTGTGACATCGATGGCACTGGT
                               TrpGlnThrTyrValAspGluHisLeuMetCysAspIleAspGlyThrGly
gi|260871354|gb|FJ715963.1|_Go  GGCAAACCTACGTTGATGAGCACTTGATGTGTGACATCGATGGCACTGGT
                               TrpGlnThrTyrValAspGluHisLeuMetCysAspIleAspGlyThrGly
gi|260871352|gb|FJ715962.1|_Go  GGCAAACCTACGTTGATGAGCACTTGATGTGTGACATCGATGGCACTGGT
                               TrpGlnThrTyrValAspGluHisLeuMetCysAspIleAspGlyThrGly

gi|217331645|gb|AY189970.2|_Go  CATCACCTTTCTGCCGCTGCTATCGTTTCTGCCGCTGCTATCGTTGGTCA
                                HisHisLeuSerAlaAlaAlaIleValSerAlaAlaAlaIleValGlyHis
gi|133925930|gb|EF143830.1|_Go  CATCACC------------------TTTCTGCCGCTGCTATCGTTGGTCA
                                HisHis                  LeuSerAlaAlaAlaIleValGlyHis
gi|133925932|gb|EF143831.1|_Go  CATCACC------------------TTTCTGCCGCTGCTATCGTTGGTCA
                                HisHis                  LeuSerAlaAlaAlaIleValGlyHis
gi|133925934|gb|EF143832.1|_Go  CATCACC------------------TTTCTGCCGCTGCTATCGTTGGTCA
                                HisHis                  LeuSerAlaAlaAlaIleValGlyHis
gi|260871354|gb|FJ715963.1|_Go  CATCACC------------------TCTCTGCCGCTGCTATCGTTGGTCA
                                HisHis                  LeuSerAlaAlaAlaIleValGlyHis
gi|260871352|gb|FJ715962.1|_Go  CATCACC------------------TTTCTGCCGCTGCTATCGTTGGTCA
                                HisHis                  LeuSerAlaAlaAlaIleValGlyHis

gi|217331645|gb|AY189970.2|_Go  CGATGGCAGTATCTGGGCTCAGAGCTCTAACTTCCCCA------------
                                 AspGlySerIleTrpAlaGlnSerSerAsnPhePro
gi|133925930|gb|EF143830.1|_Go  CGATGGCAGTATCTGGGCTCAGAGCTCTAACTTCCCCAAGGTCATGATCA
                                 AspGlySerIleTrpAlaGlnSerSerAsnPheProLysValMetIle
gi|133925932|gb|EF143831.1|_Go  TGATGGCAGTATCTGGGCTCAGAGCTCTAACTTTCCCAAGGTCATGATCA
                                 AspGlySerIleTrpAlaGlnSerSerAsnPheProLysValMetIle
gi|133925934|gb|EF143832.1|_Go  TGATGGCAGTATCTGGGCTCAGAGCTCTAACTTTCCCAAGGTCATGATCA
                                 AspGlySerIleTrpAlaGlnSerSerAsnPheProLysValMetIle
gi|260871354|gb|FJ715963.1|_Go  TGATGGCAGTATCTGGGCTCAGAGCTCTAACTTTCCCAAGGTCATGATCA
                                 AspGlySerIleTrpAlaGlnSerSerAsnPheProLysValMetIle
gi|260871352|gb|FJ715962.1|_Go  CGATGGCAGTATCTGGGCTCAGAGCTCTAACTTCCCCAAGGTCATGCTCA
                                 AspGlySerIleTrpAlaGlnSerSerAsnPheProLysValMetLeu

gi|217331645|gb|AY189970.2|_Go  --------------------------------------------------

gi|133925930|gb|EF143830.1|_Go  CTCCTCTTTCATTATTTATTCCCCATCTTAGATCATCATCATCGTTATCT
                               ThrProLeuSerLeuPheIleProHisLeuArgSerSerSerSerLeuSer
```

```
gi|260871354|gb|FJ715963.1|_Go  CTCCTCTTTCATTATTTATTCCCCGTCTTAGATCATCATCATCGTTATCT
                                ThrProLeuSerLeuPheIleProArgLeuArgSerSerSerSerLeuSer
gi|260871352|gb|FJ715962.1|_Go  CTACTCTTTCATTATTTATTCCCCATCTTAGATCATCATCATCGTTATCT
                                ThrThrLeuSerLeuPheIleProHisLeuArgSerSerSerSerLeuSer

gi|217331645|gb|AY189970.2|_Go  -----------------------------AGTGTCAGCCCAAGGAGATCA
                                LysCysGlnProLysGluIle
gi|133925930|gb|EF143830.1|_Go  GGTTTAAGTTTTCCTTTGGTTTCGATTGCAGTGTCAGCCCAAGGAGATCA
                                GlyLeuSerPheProLeuValSerIleAlaValSerAlaGlnGlyAspHis
gi|133925932|gb|EF143831.1|_Go  GGTTTAAGTTTTCCTTTGGTTTCGATTGCAGTGTCAGCCCAAGGAGATCA
                                GlyLeuSerPheProLeuValSerIleAlaValSerAlaGlnGlyAspHis
gi|133925934|gb|EF143832.1|_Go  GGTTTAAGTTTTCCTTTGGTTTCGATTGCAGTGTCAGCCCAAGGAGATCA
                                GlyLeuSerPheProLeuValSerIleAlaValSerAlaGlnGlyAspHis
gi|260871354|gb|FJ715963.1|_Go  GGTTTAAGTTTTCCTTTGGTTTCGATTGCAGTGTCAGCCCAAGGAGATCA
                                GlyLeuSerPheProLeuValSerIleAlaValSerAlaGlnGlyAspHis
gi|260871352|gb|FJ715962.1|_Go  GGTTTAAGTTTTCCTTTGGTTTCGATTGCAGTGTCAGCCCAAGGAGATCA
                                GlyLeuSerPheProLeuValSerIleAlaValSerAlaGlnGlyAspHis

gi|217331645|gb|AY189970.2|_Go  CTGACATCATGAAAGATTTCGACGAACCAGGTCACCTTGCCCCTACAGGC
                                ThrAspIleMetLysAspPheAspGluProGlyHisLeuAlaProThrGly
gi|133925930|gb|EF143830.1|_Go  CTGACATCATGAAAGATTTCGACGAACCAGGTCACCTTGCCCCTACAGGC
                                EndHisHisGluArgPheArgArgThrArgSerProCysProTyrArg
gi|133925932|gb|EF143831.1|_Go  CTGACATCATGAAAGATTTCGACGAACCAGGTCACCTTGCCCCTACAGGC
                                EndHisHisGluArgPheArgArgThrArgSerProCysProTyrArg
gi|133925934|gb|EF143832.1|_Go  CTGACATCATGAAAGATTTCGACGAACCAGGTCACCTTGCCCCTACAGGC
                                EndHisHisGluArgPheArgArgThrArgSerProCysProTyrArg
gi|260871354|gb|FJ715963.1|_Go  CTGACATCATGAAAGATTTCGACGAACCAGGTCACCTTGCCCCTACAGGC
                                EndHisHisGluArgPheArgArgThrArgSerProCysProTyrArg
gi|260871352|gb|FJ715962.1|_Go  CTGACATCATGAAAGATTTCGACGAACCAGGCCACCTTGCCCCCACAGGC
                                EndHisHisGluArgPheArgArgThrArgProProCysProHisArg

gi|217331645|gb|AY189970.2|_Go  TTGCACCTTGGTGGCGCAAAGTTTATGGTCATTCAGGGTGAGCCTGGTGC
                                LeuHisLeuGlyGlyAlaLysPheMetValIleGlnGlyGluProGlyAla
gi|133925930|gb|EF143830.1|_Go  TTGCACCTTGGTGGCGCAAAGTTTATGGTCATTCAGGGTGAGCCTGGTGC
                                LeuAlaProTrpTrpArgLysValTyrGlyHisSerGlyEndAlaTrpCys
gi|133925932|gb|EF143831.1|_Go  TTGCACCTTGGTGGCGCAAAGTTTATGGTCATTCAGGGTGAGCCTGGTGC
                                LeuAlaProTrpTrpArgLysValTyrGlyHisSerGlyEndAlaTrpCys
gi|133925934|gb|EF143832.1|_Go  TTGCACCTTGGTGGCGCAAAGTTTATGGTCATTCAGGGTGAGCCTGGTGC
                                LeuAlaProTrpTrpArgLysValTyrGlyHisSerGlyEndAlaTrpCys
gi|260871354|gb|FJ715963.1|_Go  TTGCACCTTGGTGGCGCAAAGTTTATGGTCATTCAGGGTGAGCCTGGTGC
                                LeuAlaProTrpTrpArgLysValTyrGlyHisSerGlyEndAlaTrpCys
gi|260871352|gb|FJ715962.1|_Go  TTGCACCTTGGTGGCGCAAAGTTTATGGTCATTCAGGGTGAGCCTGGTGC
                                LeuAlaProTrpTrpArgLysValTyrGlyHisSerGlyEndAlaTrpCys

gi|217331645|gb|AY189970.2|_Go  TGTCATTCGTGGAAAAA---------------------------------
                                ValIleArgGlyLys
gi|133925930|gb|EF143830.1|_Go  TGTCATTCGTGGAAAAAAGGTGATTATAT----ATACATGCATGCTAGCT
                                CysHisSerTrpLysLysGlyAspTyrIle    TyrMetHisAlaSer
gi|133925932|gb|EF143831.1|_Go  TGTCATTCGTGGAAAAAAGGTGATTATAT----ATACATGCATGCTAGCT
                                CysHisSerTrpLysLysGlyAspTyrIle    TyrMetHisAlaSer
gi|133925934|gb|EF143832.1|_Go  TGTCATTCGTGGAAAAAAGGTGATTATATATACATACATGCATGCTAGCT
                                CysHisSerTrpLysLysGlyAspTyrIleTyrIleHisAlaCysEndLeu
gi|260871354|gb|FJ715963.1|_Go  TGTCATTCGTGGAAAAAAGGTGATTATATATACATACATGCATGCTAGCT
                                CysHisSerTrpLysLysGlyAspTyrIleTyrIleHisAlaCysEndLeu
gi|260871352|gb|FJ715962.1|_Go  TGTCATTCGTGGAAAAAAGGTGATTATAT----ATACATGCATGCTAGCT
                                CysHisSerTrpLysLysGlyAspTyrIle    TyrMetHisAlaSer

gi|217331645|gb|AY189970.2|_Go  --------------------------------------------------

gi|133925930|gb|EF143830.1|_Go  TTAATATATTATACTACAAAAAATCTAAAAAAAC--TGTGTGTTTTAATA
                                PheAsnIleLeuTyrTyrLysLysSerLysLysThr  ValCysPheAsn
gi|133925932|gb|EF143831.1|_Go  TTAATATATTATACTACAAAAAATCTAAAAAAAC--TGTGTGTTTTAATA
                                PheAsnIleLeuTyrTyrLysLysSerLysLysThr  ValCysPheAsn
gi|133925934|gb|EF143832.1|_Go  TTAATATATTATACTAGAAAAAATCTCAAAAAACTGTGTGTGTTGTAATA
                                EndTyrIleIleLeuGluLysIleSerLysAsnCysValCysCysAsn
gi|260871354|gb|FJ715963.1|_Go  TTAATATATTATACTAGAAAAAATCTCAAAAAACTGTGTGTGTTGTAATA
                                EndTyrIleIleLeuGluLysIleSerLysAsnCysValCysCysAsn
gi|260871352|gb|FJ715962.1|_Go  TTAATATATTATACTACAAAAAATCTCAAAAAACTGTGTGTGTTTTAACA
                                PheAsnIleLeuTyrTyrLysLysSerGlnLysThrValCysValLeuThr
```

```
gi|217331645|gb|AY189970.2|_Go   -------------AGGGATCTGGAGGGGTGACTATTAAGAAAACAGCACAA
                                              LysGlySerGlyGlyValThrIleLysLysThrAlaGln
gi|133925930|gb|EF143830.1|_Go   TATGATGTGGGCAGGGATCTGGAGGGGTGACTATTAAGAAAACAGCACAA
                                IleEndCysGlyGlnGlySerGlyGlyValThrIleLysLysThrAlaGln
gi|133925932|gb|EF143831.1|_Go   TATGACGTGGGCAGGGATCTGGAGGGGTGACTATTAAGAAAACAGCACAA
                                IleEndArgGlyGlnGlySerGlyGlyValThrIleLysLysThrAlaGln
gi|133925934|gb|EF143832.1|_Go   TATGACGTGGGCAGGGATCTGGAGGGGTGACTATTAAGAAAACAGCACAA
                                IleEndArgGlyGlnGlySerGlyGlyValThrIleLysLysThrAlaGln
gi|260871354|gb|FJ715963.1|_Go   TATGACGTGGGCAGGGATCTGGAGGGGTGACTATTAAGAAAACAGCACAA
                                IleEndArgGlyGlnGlySerGlyGlyValThrIleLysLysThrAlaGln
gi|260871352|gb|FJ715962.1|_Go   TATGATGTGGGCAGGGATCTGGAGGGGTGACTATTAAGAAAACAGCACAA
                                 TyrAspValGlyArgAspLeuGluGlyEndLeuLeuArgLysGlnHisLys

gi|217331645|gb|AY189970.2|_Go   GCACTTGTGTTTGGGATATATGAAGAACCAGTGACTCCGGGGCAATGCAA
                                 AlaLeuValPheGlyIleTyrGluGluProValThrProGlyGlnCysAsn
gi|133925930|gb|EF143830.1|_Go   GCACTTGTGTTTGGGATATATGAAGAACCAGTGACTCCGGGGCAATGCAA
                                 AlaLeuValPheGlyIleTyrGluGluProValThrProGlyGlnCysAsn
gi|133925932|gb|EF143831.1|_Go   GCACTTGTGTTTGGGATATATGAAGAACCAGTGACTCCGGGGCAATGCAA
                                 AlaLeuValPheGlyIleTyrGluGluProValThrProGlyGlnCysAsn
gi|133925934|gb|EF143832.1|_Go   GCACTTGTGTTTGGGATATATGAAGAACCAGTGACTCCGGGGCAATGCAA
                                 AlaLeuValPheGlyIleTyrGluGluProValThrProGlyGlnCysAsn
gi|260871354|gb|FJ715963.1|_Go   GCACTTGTGTTTGGGATATATGAAGAACCAGTGACTCCGGGGCAATGCAA
                                 AlaLeuValPheGlyIleTyrGluGluProValThrProGlyGlnCysAsn
gi|260871352|gb|FJ715962.1|_Go   GCACTTGTGTTTGGGATATATGAAGAACCAGTGACTCCGGGGCAATGCAA
                                  HisLeuCysLeuGlyTyrMetLysAsnGlnEndLeuArgGlyAsnAla

gi|217331645|gb|AY189970.2|_Go   CATGGTTGTGGAGAGGTTGGGCGATTATCTTGCAGAACAGGGCCTGTAGT
                                  MetValValGluArgLeuGlyAspTyrLeuAlaGluGlnGlyLeuEnd
gi|133925930|gb|EF143830.1|_Go   CATGGTTGTGGAGAGGTTGGGCGATTATCTTGCAGAACAGGGCCTGTAGT
                                  MetValValGluArgLeuGlyAspTyrLeuAlaGluGlnGlyLeuEnd
gi|133925932|gb|EF143831.1|_Go   CATGGTTGTGGAGAGGTTGGGCGATTATCTTGCAGAACAGGGCCTGTAGT
                                  MetValValGluArgLeuGlyAspTyrLeuAlaGluGlnGlyLeuEnd
gi|133925934|gb|EF143832.1|_Go   CATGGTTGTGGAGAGGTTGGGCGATTATCTTGCAGAACAGGGCCTGTAGT
                                  MetValValGluArgLeuGlyAspTyrLeuAlaGluGlnGlyLeuEnd
gi|260871354|gb|FJ715963.1|_Go   CATGGTTGTGGAGAGGTTGGGCGATTATCTTGCAGAACAGGGCCTGTAGT
                                  MetValValGluArgLeuGlyAspTyrLeuAlaGluGlnGlyLeuEnd
gi|260871352|gb|FJ715962.1|_Go   CATGGTTGTGGAGAGGTTGGGCGATTATCTTGCAGAACAGGGCCTGTAGT
                                ThrTrpLeuTrpArgGlyTrpAlaIleIleLeuGlnAsnArgAlaCysSer

gi|217331645|gb|AY189970.2|_Go   CGACCACCTAGCTTTTCATATTCAATGCCTTAATTCCCCCATATGTTTTT
                                SerThrThrEndLeuPheIlePheAsnAlaLeuIleProProTyrValPhe
gi|133925930|gb|EF143830.1|_Go   CGA-----------------------------------------------
                                Ser
gi|133925932|gb|EF143831.1|_Go   CGA-----------------------------------------------
                                Ser
gi|133925934|gb|EF143832.1|_Go   CGA-----------------------------------------------
                                Ser
gi|260871354|gb|FJ715963.1|_Go   CGA-----------------------------------------------
                                Ser
gi|260871352|gb|FJ715962.1|_Go   CGA-----------------------------------------------
                                 Arg

gi|217331645|gb|AY189970.2|_Go   GTTTCTTCTTTTTTAAGTCTTGGATTCCGAGTAATTTGCGTTTTCTTCCA
                                 ValSerSerPheLeuSerLeuGlyPheArgValIleCysValPhePheHis
gi|133925930|gb|EF143830.1|_Go   --------------------------------------------------
gi|133925932|gb|EF143831.1|_Go   --------------------------------------------------
gi|133925934|gb|EF143832.1|_Go   --------------------------------------------------
gi|260871354|gb|FJ715963.1|_Go   --------------------------------------------------
gi|260871352|gb|FJ715962.1|_Go   --------------------------------------------------

gi|217331645|gb|AY189970.2|_Go   TTGAAAAACTATATACGGTTTGATAAGATGGTTTGACTGGCATTCGGACT
                                  EndLysThrIleTyrGlyLeuIleArgTrpPheAspTrpHisSerAsp
gi|133925930|gb|EF143830.1|_Go   --------------------------------------------------
```

```
                                         EndLysThrIleTyrGlyLeuIleArgTrpPheAspTrpHisSerAsp
gi|133925930|gb|EF143830.1|_Go   --------------------------------------------------
gi|133925932|gb|EF143831.1|_Go   --------------------------------------------------
gi|133925934|gb|EF143832.1|_Go   --------------------------------------------------
gi|260871354|gb|FJ715963.1|_Go   --------------------------------------------------
gi|260871352|gb|FJ715962.1|_Go   --------------------------------------------------

gi|217331645|gb|AY189970.2|_Go   TGGGTTGATCTGAGTTGTTTTCTTTTCGCCATATCCGTTTGTTTTGTTTT
                                LeuGlyEndSerGluLeuPheSerPheArgHisIleArgLeuPheCysPhe
gi|133925930|gb|EF143830.1|_Go   --------------------------------------------------
gi|133925932|gb|EF143831.1|_Go   --------------------------------------------------
gi|133925934|gb|EF143832.1|_Go   --------------------------------------------------
gi|260871354|gb|FJ715963.1|_Go   --------------------------------------------------
gi|260871352|gb|FJ715962.1|_Go   --------------------------------------------------

gi|217331645|gb|AY189970.2|_Go   CTTAATTTTTCGTTCTTGGGTTCTGTTTGTATTTATAATCCCCCCATCAG
                                 LeuAsnPheSerPheLeuGlySerValCysIleTyrAsnProProIleSer
gi|133925930|gb|EF143830.1|_Go   --------------------------------------------------
gi|133925932|gb|EF143831.1|_Go   --------------------------------------------------
gi|133925934|gb|EF143832.1|_Go   --------------------------------------------------
gi|260871354|gb|FJ715963.1|_Go   --------------------------------------------------
gi|260871352|gb|FJ715962.1|_Go   --------------------------------------------------

gi|217331645|gb|AY189970.2|_Go   CCATCAAGTGGATTGCGGGGGTTTTTGNTTTTTNGGGTTTTATTTTTTTC
                                   HisGlnValAspCysGlyGlyPheXXXPheXXXGlyPheTyrPhePhe
gi|133925930|gb|EF143830.1|_Go   --------------------------------------------------
gi|133925932|gb|EF143831.1|_Go   --------------------------------------------------
gi|133925934|gb|EF143832.1|_Go   --------------------------------------------------
gi|260871354|gb|FJ715963.1|_Go   --------------------------------------------------
gi|260871352|gb|FJ715962.1|_Go   --------------------------------------------------

gi|217331645|gb|AY189970.2|_Go   CTTTTTGGGTGGCACCTATTTTGGATGCCCACCTATAT
                                ProPheTrpValAlaProIleLeuAspAlaHisLeuTyr
gi|133925930|gb|EF143830.1|_Go   --------------------------------------
gi|133925932|gb|EF143831.1|_Go   --------------------------------------
gi|133925934|gb|EF143832.1|_Go   --------------------------------------
gi|260871354|gb|FJ715963.1|_Go   --------------------------------------
gi|260871352|gb|FJ715962.1|_Go   --------------------------------------
```
