# Supplementary material for: Domestication-driven Gossypium profilin 1 (GhPRF1) gene transduces early flowering phenotype in tobacco by spatial alteration of apical/floral-meristem related gene expression
Source: BMC Plant Biol. 2016 May 13;16:112. doi: 10.1186/s12870-016-0798-0 (PMC4866011; doi:10.1186/s12870-016-0798-0)
Supplement: Additional file 10: — Primer sequences used in the study. (PPT 207 kb) [file 12870_2016_798_MOESM10_ESM.ppt]

## Slide 1
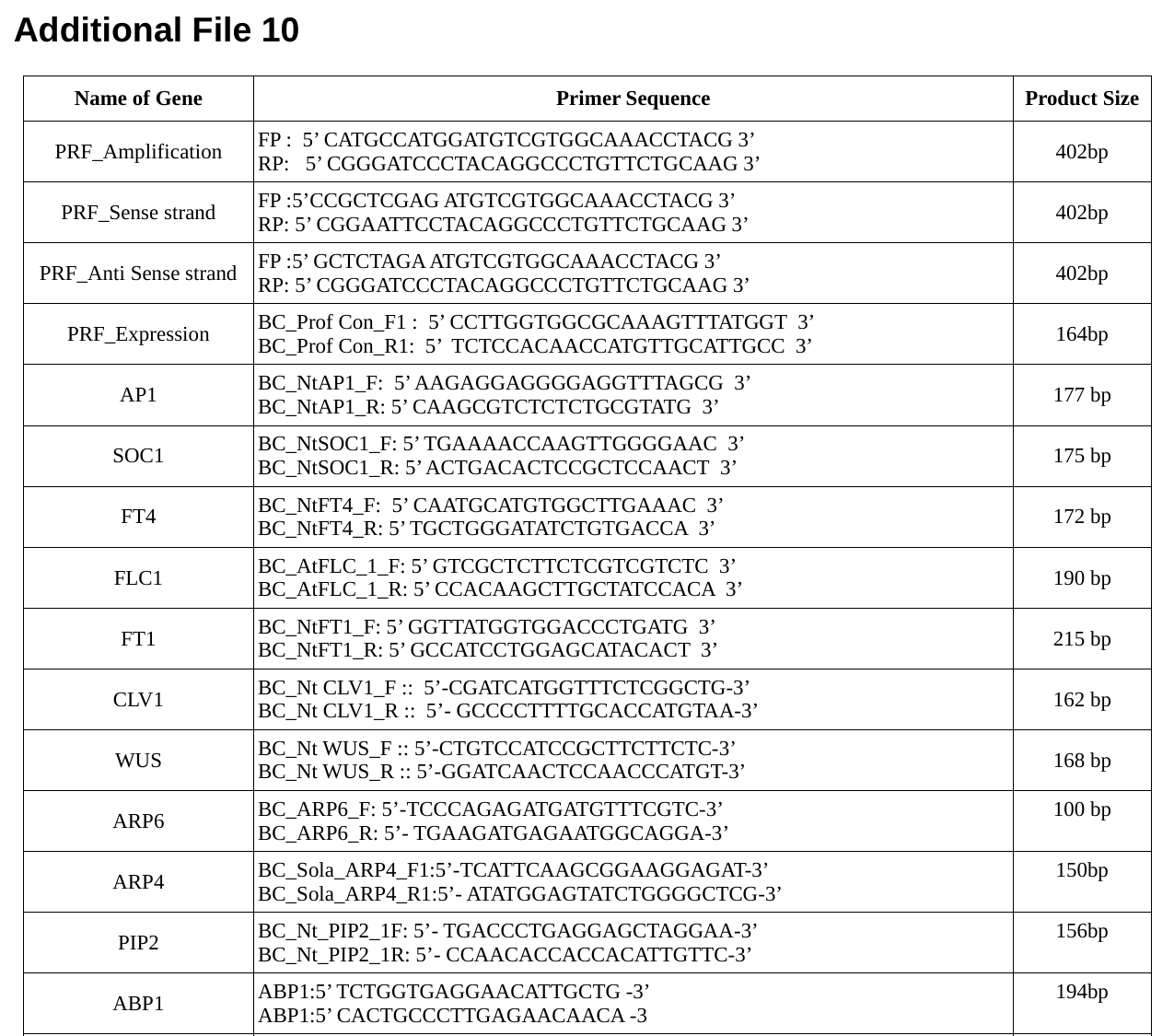

Additional File 10
| Name of Gene | Primer Sequence | Product Size |
| --- | --- | --- |
| PRF\_Amplification | FP : 5’ CATGCCATGGATGTCGTGGCAAACCTACG 3’ RP: 5’ CGGGATCCCTACAGGCCCTGTTCTGCAAG 3’ | 402bp |
| PRF\_Sense strand | FP :5’CCGCTCGAG ATGTCGTGGCAAACCTACG 3’ RP: 5’ CGGAATTCCTACAGGCCCTGTTCTGCAAG 3’ | 402bp |
| PRF\_Anti Sense strand | FP :5’ GCTCTAGA ATGTCGTGGCAAACCTACG 3’ RP: 5’ CGGGATCCCTACAGGCCCTGTTCTGCAAG 3’ | 402bp |
| PRF\_Expression | BC\_Prof Con\_F1 : 5’ CCTTGGTGGCGCAAAGTTTATGGT 3’ BC\_Prof Con\_R1: 5’ TCTCCACAACCATGTTGCATTGCC 3’ | 164bp |
| AP1 | BC\_NtAP1\_F:  5’ AAGAGGAGGGGAGGTTTAGCG  3’ BC\_NtAP1\_R: 5’ CAAGCGTCTCTCTGCGTATG  3’ | 177 bp |
| SOC1 | BC\_NtSOC1\_F: 5’ TGAAAACCAAGTTGGGGAAC  3’ BC\_NtSOC1\_R: 5’ ACTGACACTCCGCTCCAACT  3’ | 175 bp |
| FT4 | BC\_NtFT4\_F:  5’ CAATGCATGTGGCTTGAAAC  3’ BC\_NtFT4\_R: 5’ TGCTGGGATATCTGTGACCA  3’ | 172 bp |
| FLC1 | BC\_AtFLC\_1\_F: 5’ GTCGCTCTTCTCGTCGTCTC  3’ BC\_AtFLC\_1\_R: 5’ CCACAAGCTTGCTATCCACA  3’ | 190 bp |
| FT1 | BC\_NtFT1\_F: 5’ GGTTATGGTGGACCCTGATG  3’ BC\_NtFT1\_R: 5’ GCCATCCTGGAGCATACACT  3’ | 215 bp |
| CLV1 | BC\_Nt CLV1\_F :: 5’-CGATCATGGTTTCTCGGCTG-3’ BC\_Nt CLV1\_R :: 5’- GCCCCTTTTGCACCATGTAA-3’ | 162 bp |
| WUS | BC\_Nt WUS\_F :: 5’-CTGTCCATCCGCTTCTTCTC-3’ BC\_Nt WUS\_R :: 5’-GGATCAACTCCAACCCATGT-3’ | 168 bp |
| ARP6 | BC\_ARP6\_F: 5’-TCCCAGAGATGATGTTTCGTC-3’ BC\_ARP6\_R: 5’- TGAAGATGAGAATGGCAGGA-3’ | 100 bp |
| ARP4 | BC\_Sola\_ARP4\_F1:5’-TCATTCAAGCGGAAGGAGAT-3’ BC\_Sola\_ARP4\_R1:5’- ATATGGAGTATCTGGGGCTCG-3’ | 150bp |
| PIP2 | BC\_Nt\_PIP2\_1F: 5’- TGACCCTGAGGAGCTAGGAA-3’ BC\_Nt\_PIP2\_1R: 5’- CCAACACCACCACATTGTTC-3’ | 156bp |
| ABP1 | ABP1:5’ TCTGGTGAGGAACATTGCTG -3’ ABP1:5’ CACTGCCCTTGAGAACAACA -3 | 194bp |
| L25 | BC\_L25\_F:  5’  CCCCTCACCACAGAGTCTGC 3’  BC\_L25\_R: 5’ AAGGGTGTTGTTGTCCTCAATCTT 3’ | 51bp |
